# Machine Learning for RNA-Targeting Drug Design

**DOI:** 10.1021/acs.jcim.6c01094

**Published:** 2026-07-14

**Authors:** Wissam Karroucha, Carlos Oliver, Véronique Stoven, Vincent Mallet

**Affiliations:** † Mines Paris, 52808 PSL Research University, CBIO, Paris 75272, France; ‡ Institut Curie, 5718 PSL Research University, Paris 75248, France; § INSERM, U1331, Paris 75654 France; ∥ Vanderbilt University, Nashville, Tennessee 37235, United States

**Keywords:** RNA, drug design, small molecule, specificity, machine learning, virtual screening

## Abstract

Targeting RNA with small molecules offers significant therapeutic potential. Machine learning could substantially accelerate preclinical drug discovery, from hit identification to lead optimization. Yet a limitation emerges: drug design machine learning models, designed for proteins, are not readily applicable to RNAs because of fundamental differences between RNAs and proteins in both structural characteristics and interactions with small molecules. RNA-specific approaches have consequently emerged, primarily focusing on binding site identification and virtual screening. In this review, we comprehensively compare machine learning tools for RNA-targeting drug design according to the tasks they address, their methodology and their relevance in RNA-specific contexts. As open challenges will catalyze new method development, we emphasize the need for standardized, drug design-specific evaluation approaches. We provide clear guidelines to establish these standards and present a benchmark assessing the ability of current machine learning models to predict specific drug–RNA interactions.

## Graphical abstract

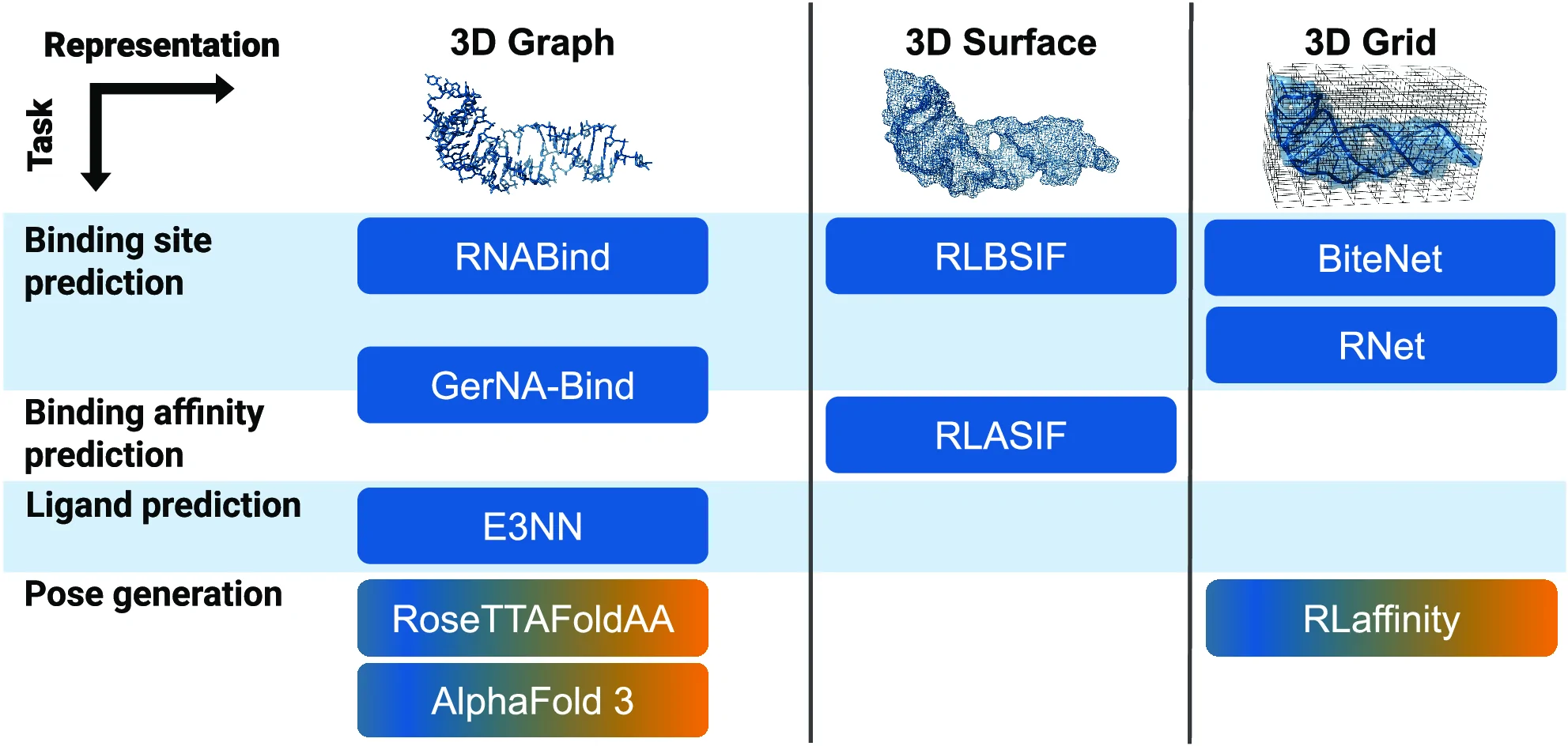

## 1 Introduction

RNA has emerged as a compelling target for small molecule therapeutics.^1,2^ Beyond the canonical role of mRNAs in genetic information transfer, noncoding RNAs play pivotal roles in gene expression regulation^3^ and are involved in numerous human disease pathways.^4^ RNAs have proven to be targetable by small molecules,^5,6^ paving the way for novel therapeutic avenues, particularly for diseases involving undruggable proteins.^7^ Despite this potential, RNAs remain significantly under-exploited as therapeutic targets. A few antibiotic families targeting rRNAs (rRNAs) successfully reached the market because these RNAs are well structured and because the prokaryotic ribosomes differ substantially from those of eukaryotes.^8^ However, the first FDA-approved drug targeting a non-ribosomal RNA (risdiplam, a pre-mRNA splicing modifier against spinal muscular atrophy) was only authorized in 2020, and remains the only one to date.

At the same time, drug design-focused machine learning models are emerging. In virtual screening especially, their computational efficiency allows for the exploration of vastly larger chemical libraries, significantly increasing the probability of identifying high-affinity compounds. This holds significant potential for accelerating RNA-targeting drug design.

However, the field has been historically dominated by protein targets, leading to the development of highly successful machine learning methods that are fundamentally protein-centric (e.g., for binding affinity prediction^9–12^ and computational docking^13–15^ ). These approaches cannot be directly applied to RNA, whose fundamental differences from proteins necessitate specialized modeling strategies.

### 1.1 On the Uniqueness of RNA

RNAs exhibit greater conformational flexibility than proteins, complicating their experimental structure determination through X-ray crystallography.^16^ These experimental challenges contribute to the scarcity of RNA structural data: as of 2025, the Protein Data Bank (PDB)^17^ contains 232,962 protein structures compared to only 8,767 RNA structures. Moreover, RNA-small molecule interactions are governed by distinct physicochemical principles. In particular, they are dominated by electrostatic interactions and π-stacking, whereas protein-small molecule interactions rely more heavily on hydrophobic contacts.^18^ Furthermore, evidence suggests that specific RNA binding sites are more polar^19^ and more deeply buried within their structures^20^ than protein binding sites.

Moreover, several RNA properties favor nonspecific interactions.^21^ Compared to the 20 amino acids composing proteins, the four nucleotides that compose RNA display limited chemical diversity. They can all form π-stacking through their bases,^7^ and electrostatic interactions because of the negatively charged backbone.^18,22^ This nonspecificity explains the high toxicity associated with most current RNA-targeting drugs. Nonspecific RNA binders include intercalators, interacting with RNAs through π-stacking and hydrophobic contacts, and aminoglycosides. For instance, Figure 1A illustrates a nonspecific RNA-aminoglycoside interaction (between ribostamycin and the 16S rRNA A-Site) that relies on many H-bonds involving atoms from the phosphate groups of the RNA (colored in red). These interactions are not sequence-specific.^23^ The applicability of such antibiotics is thus limited to critical scenarios or to localized, nonabsorbed treatments e.g., on skin.

**Figure 1:**
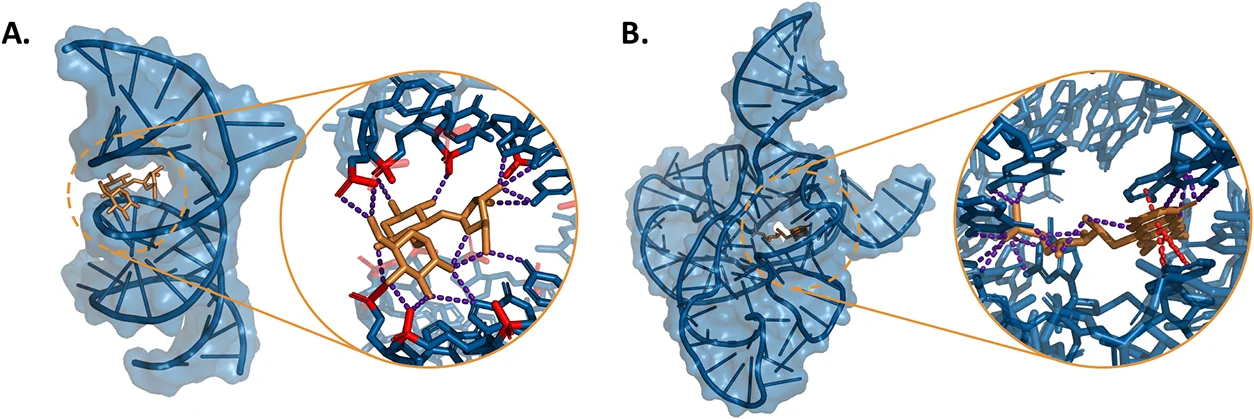
Structures of nonspecific (**A**) and specific (**B**) RNA-small molecule binding sites. Purple dashed lines represent H bonds and red dashed lines represent π-stacking. Figures were generated using PyMol^25^ and fingeRNAt.^26^ **A.** Structure of the 16S-RRNA A-Site unspecifically bound to Ribostamycin (PDB 2et5). **B.** Flavin mononucleotide (FMN) riboswitch bound to FMN (PDB 2yie).

However, some RNAs display specific binding to small molecules, especially through hydrogen bonds involving RNA bases. For instance, the Flavin mononucleotide (FMN) riboswitch, which binds specifically to the FMN molecule, displays a highly buried binding site (Figure 1B).^7^ The ligand engages with nucleotides across five different loops. Moreover, these interactions involve the Watson–Crick faces of some nucleobases, which are known to form highly specific bonds.^24^ These complex contacts spanning multiple loops result in a very specific RNA-ligand interaction. Achieving such specific binding to RNA targets would open new therapeutic avenues.

Given these fundamental differences between RNA and protein targets, the development of RNA-specific machine learning approaches is essential. The rapid emergence of novel methods across diverse tasks in the drug discovery pipeline, employing varied modeling approaches and data sets, necessitates a systematic organization of the field based both on the task and on the methodological approach.

### 1.2 Preclinical Drug Discovery Pipeline

The preclinical drug discovery pipeline comprises several steps (Figure 2A). First, a target (in our case, an RNA involved in the pathology to be treated) must be identified and validated (*Target identification and validation*).

**Figure 2:**
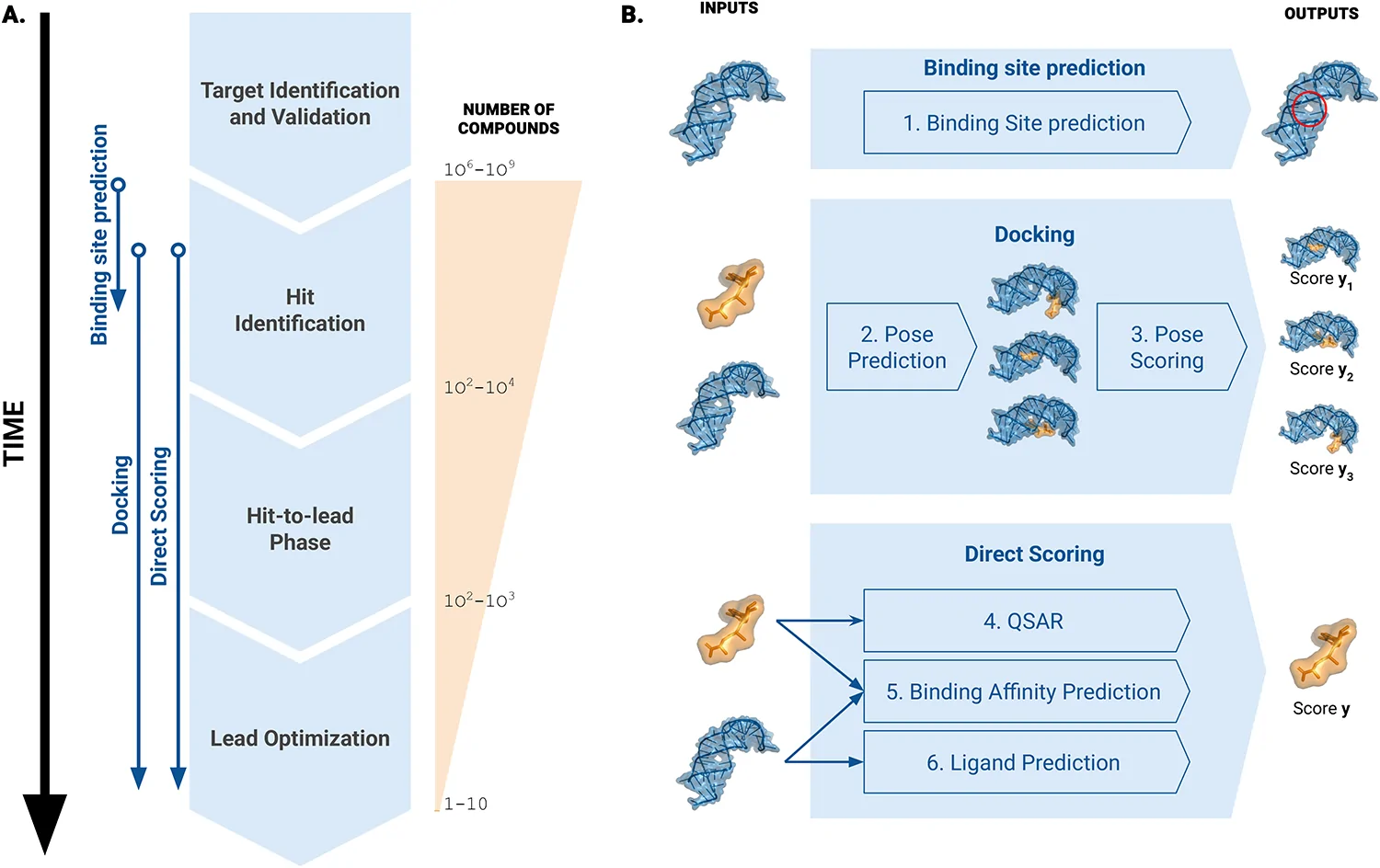
Overview of the preclinical drug discovery pipeline (**A**) along with six machine learning tasks (**B**) assisting it. Binding site prediction (1) enables researchers to focus on subparts of an RNA target. Docking, resulting from the joint use of the pose generation (2) and pose scoring (3) tasks, outputs the bound structure of an RNA-ligand pair along with a binding propensity score. Direct scoring methods (QSAR (4), Binding Affinity Prediction (5) and Ligand Prediction (6)) provide a binding propensity score without predicting the complex structure. They differ by the input data they use.

Once a target has been validated, *Hit identification* focuses on identifying promising small molecules (hits) that show demonstrated affinity to this target.^27^ A common approach to hit identification relies on screening campaigns, which assess the ability of molecules from a chemical library to bind to the target. However, the drug-like chemical space is so vast (between 10^20^ and 10^24^ molecules up to 30 atoms^28^) that it cannot be experimentally tested, calling for efficient in-silico methods.

The pipeline continues through two key refinement stages. During the *Hit-to-lead phase*, hits are chemically refined to significantly increase their affinity and selectivity, generally reaching nanomolar affinity; the resulting compounds are called lead compounds.^27^ During *Lead optimization*, lead compound structures are further refined to optimize their pharmacological profile, including properties such as synthesizability, delivery, and reduced side-effects, while preserving high affinity and selectivity. Successful compounds become preclinical drug candidates, ready to enter the translational investigation stage.

### 1.3 Overview

This review systematically surveys machine learning methods for RNA-targeting drug design, focusing specifically on the stages spanning from hit identification to hit-to-lead optimization. Our discussion is organized around four key questions:

- *What are the contributions of machine learning to RNA drug design?* Section 2 comprehensively categorizes existing methods by the specific drug design problem they address and identifies research gaps.
- *What are the possible machine learning methods for RNA data?* We examine the possible mathematical representations and machine learning encodings of RNAs that can be used to solve the above tasks, and use this perspective to dissect existing literature in Section 3.
- *What data are available and should be used to train our models?* We discuss best practices for data collection and partitioning, along with existing benchmarking data sets in Section 4.
- *How do we rigorously assess model performance?* Our final Section 5 reviews model evaluation in drug design contexts and highlights crucial pitfalls. We notably investigate a critical yet underexplored question in RNA binding affinity prediction: whether current models genuinely capture specific RNA-small molecule interactions or instead rely primarily on ligand features. We propose a new performance assessment and use it to compare four state-of-the-art models.

Our analysis serves a dual purpose: to guide researchers in RNA drug design toward the most suitable computational approaches for their work and to offer the machine learning community insights into design principles and research gaps in machine learning for RNA-targeting drug design.

## 2 Drug Design Problems Addressed

Machine learning can contribute to drug design in several ways throughout the preclinical drug discovery pipeline. In target identification, machine learning can help to elucidate RNA functions and regulatory pathways.^29,30^ This review focuses specifically on later stages, once a target has been identified. Machine learning models can be used to predict the location of potential binding sites on the target. In hit-to-lead and lead optimization, models predict the structure of the RNA-ligand complex (RNA-ligand pose), thus facilitating rational and structure-based drug design: the knowledge of the pose enables chemists to identify possible and suitable structural refinements.^1^

Moreover, machine learning’s key contribution to preclinical drug discovery is small molecule scoring, which consists of assigning a score to a small molecule assessing its properties. Binding scores are relevant to several stages of the drug discovery pipeline. Indeed, during hit identification, small molecule scoring allows researchers to perform virtual screening: they rank compounds based on their scores, thus focusing further experimental assays on the best-ranked candidates. During hit-to-lead and lead optimization, binding scores enable chemists to anticipate the impact of molecular optimization on binding. While machine learning models also exist for predicting other properties, such as pharmacokinetics or toxicity, these are beyond the scope of this review; we refer the reader to specialized works.^31,32^

### 2.1 Small Molecule Scoring Formulation

In the present paper, binding propensity is evaluated according to a score denoted *y*. Depending on the application, *y* may represent either a binary indicator of binding or a continuous variable. A binary indicator allows for the exploitation of nonquantitative data, originating from wet-lab assays such as small molecule microarrays,^33^ whereas a continuous variable accounts for finer-grained differences in binding energy.

During the hit identification stage, small molecule scoring aims to discriminate between active compounds and decoys (presumed nonbinders) to prefilter the chemical library. Therefore, a binary score *y* is most often used. Conversely, when used during hit-to-lead and lead optimization stages, small molecule scoring must guide fine structural modifications, for which even subtle binding affinity differences become important, therefore, a continuous score *y* is preferred. The most common choice is the binding affinity, defined as the negative logarithm (p*K*_d_) of the dissociation constant (*K*_d_) of the RNA-ligand complex.

### 2.2 Which Drug Design Tasks can Machine Learning Perform?

Machine learning methods for RNA-targeting drug design can be categorized into three families, themselves broken down into six tasks (Figure 2B).

The first family consists of a single task: *binding site prediction* (1), which involves identifying potential binding sites on RNA targets. This step typically occurs during hit identification, helping researchers focus on specific binding sites rather than the whole target during later stages (small molecule scoring and pose generation), thus facilitating these problems.

The second family consists of *docking approaches*, which focus on predicting the target-ligand pose. This family includes *pose generation* (2) (predicting RNA-ligand 3D poses) and *pose scoring* (3) (predicting binding propensity from a pose).

The third family gathers methods that estimate RNA-small molecule binding propensity without inferring the pose, which we call *direct scoring* methods. Direct scoring can be formulated as three distinct tasks: *Quantitative structure–activity relationship* (QSAR) (4), which only takes a ligand as input, *ligand prediction* (5), which only takes an RNA target as input, and *binding affinity prediction* (6), which takes both a ligand and an RNA target as input.

Figure 3 and Tables in Appendix A provide a comprehensive mapping from these tasks to existing computational methods. This mapping helps identify the most relevant approaches for a specific drug design problem.

**Figure 3:**
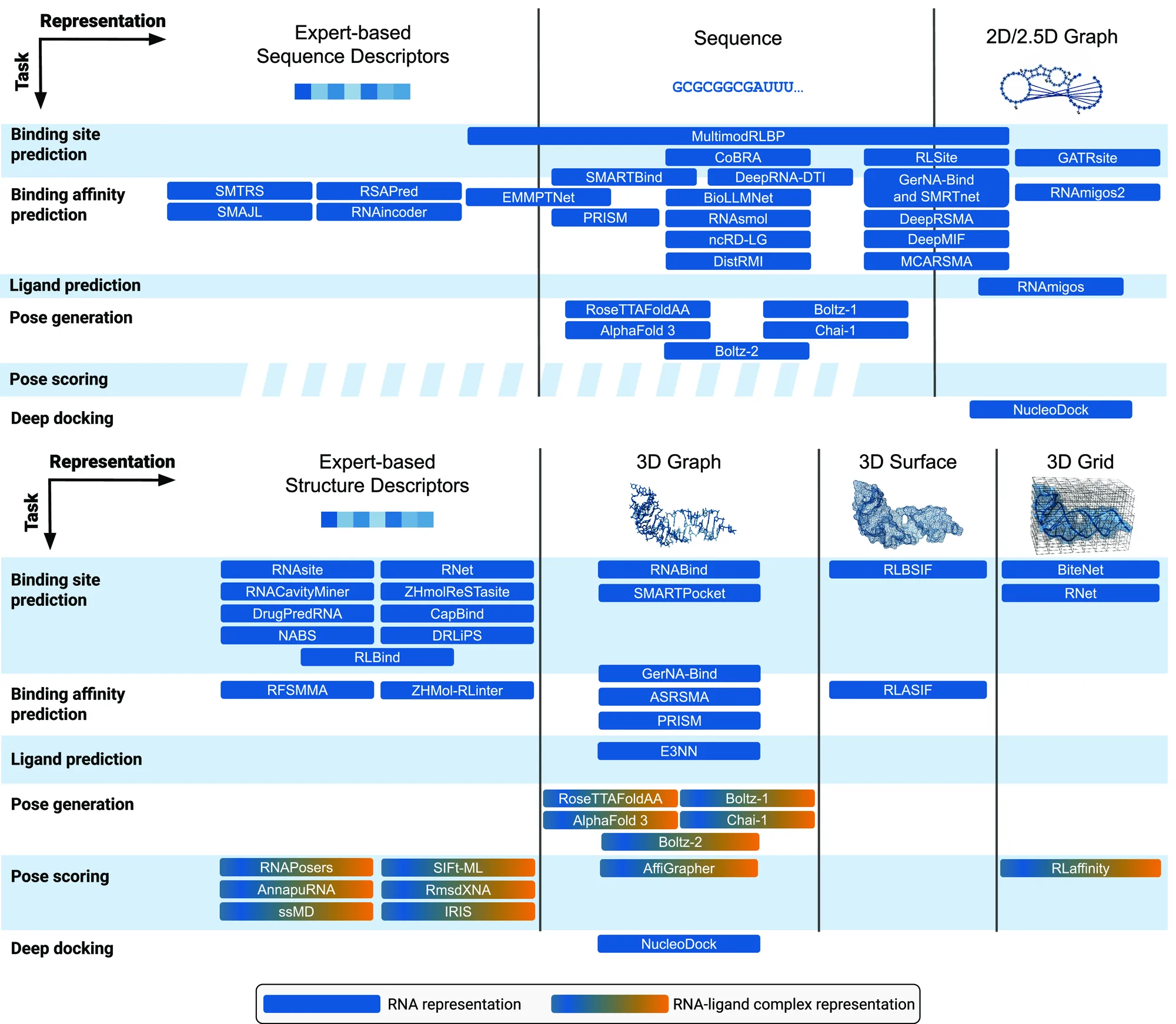
Overview of machine learning methods for RNA-targeting drug design. Methods are categorized according to the RNA and complex mathematical representations they rely on (columns). The upper table represents methods relying on sequence-based RNA representations and the lower one represents methods relying on structure-based RNA representations. RNA representations are depicted by blue boxes and RNA-ligand complex representations are represented as blue/orange boxes. Methods are also placed according to the drug discovery task they perform (rows), from hit identification to lead optimization. Note that the QSAR task is not represented in this figure since it does not involve an RNA representation.

### 2.3 Binding Site Prediction

In binding site prediction, the input is an RNA *R* and the output is a nucleotide-level binary annotation identifying the nucleotides participating in a binding site. This prediction allows subsequent drug discovery efforts to focus on specific binding site structures, thereby improving their efficiency. A ligand-specific variant formulation was proposed in DeepRNA-DTI^34^ and,^35–37^ taking a ligand as an extra-input to predict its specific binding-site. While this removes the assumption that all ligands would have the same binding site, this formulation complicates the application of independent binding site-focused methods, such as docking. The emergence of *blind docking* and *cofolding* approaches, which jointly predict RNA-ligand poses and binding sites from complete RNA structures, might question the need for binding site prediction. However, predicting the binding site before running a docking or cofolding model is computationally lighter and often more accurate.^38^

Classical computational methods for binding site prediction are structure-based since binding sites have an intrinsically geometric signature. Early computational approaches for RNA-small molecule binding site prediction, including Rsite,^39^ Rsite2,^40^ and RBind,^41^ relied on handcrafted statistical thresholds. Machine learning methods have subsequently been developed to identify RNA binding sites. The first machine learning approaches to binding site prediction^42–48^ relied on expert-based chemical, geometric or sequence-based features, combined with shallow machine learning models.

To go beyond handcrafted features, recent methods leverage geometric deep learning models to learn directly from the raw 3D data, using graph,^49–53^ surface^54^ or grid^55,56^ representations. These methods are the current best performers, with SMARTPocket^53^ reaching the highest AUC, followed by GATRSite^52^ and MultimodRLBP,^49^ and RLBSIF^54^ reaching the highest Matthews correlation coefficient on the widely used TR60 and TE18 data sets introduced in RNASite.^42^ BiteNet^55^ also shows competitive performance but on a different data set, preventing fair comparison with its most recent competitors.

However, structure-based approaches face a critical limitation: the limited number of experimentally resolved RNA-small molecule complexes. Two approaches have been proposed in the literature to overcome this issue. First, RNet^56^ and SMARTPocket^53^ proposed a transfer learning approach, leveraging the more abundant protein–ligand structural data to identify RNA binding sites. Alternatively, building on recent advances in RNA language models, the latest approaches MultiModRLBP,^49^ RLsite,^51^ and CoBRA^57^ include RNA language models in their architectures. By leveraging a large amount of sequence data, RNA language models may compensate for the limited amount of structural data. This additional data brings information regarding coevolution, homologues and sequence patterns. Since structural information can be implicitly encoded in a language model,^58^ CoBRA^57^ goes even further in this direction by only relying on an RNA language model, completely bypassing structure.

Another limitation of existing binding site prediction approaches is that they are trained on *holo* (ligand-bound) RNA structures. This limits their applicability, since experimental researchers only have *apo* (unbound) structures at their disposal. RNA conformational flexibility makes the development of *apo*-based models more challenging than for proteins. Interestingly, despite not explicitly incorporating dynamics, SMARTPocket^53^ demonstrates an ability to generalize to *apo* structures and identify cryptic pockets. However, this only holds when the conformational rearrangements remain limited. To address this challenge, Molearn^59^ proposes a promising approach: a variational autoencoder (VAE; a family of generative models) generates *holo* conformations of RNAs from *apo* conformations of the same RNAs. Such models could be a stepping stone for *apo*-based models, and be used to build a data set containing multiple conformations of the same RNAs.

### 2.4 Docking

#### 2.4.1 Pose Generation

The *pose generation* task takes an RNA (or RNA pocket) *R* and a ligand *L* as input and outputs the 3D structure of the complex *C* formed by *R* and *L*. The pose generation task can be formulated in two contexts: site-specific docking (*R* is a pocket), where the binding site is predefined and only local search for poses is required, and blind docking (*R* is a whole RNA), where binding site identification and pose generation must be performed simultaneously.

Existing docking software such as DOCK,^60^ AutoDock,^61^ and AutoDock Vina,^62^ were primarily designed for protein–ligand pose generation and rely on physical force fields optimized for protein structures. Adaptations to RNA systems include modified versions of established tools such as DOCKing,^63^ AutoDock,^64^ and ICM.^65^ Additionally, RNA-specific docking tools have been developed, including MORDOR,^66^ rDock,^67^ RLDock,^68^ and RLDOCKScore.^69^

A limitation of traditional docking is the computational cost of sampling poses during the pose generation step. To limit this cost, a widely used simplifying assumption is to consider the receptor to be rigid, which is a particularly strong assumption in the case of RNA. Traditional force fields were also shown to have unstable performance and limited accuracy for pose scoring.^70^ Machine learning methods can be used to address these limitations and enhance the docking pipeline, primarily by performing pose generation through two distinct approaches: *deep docking*, relying on a structure-based RNA representation, and *cofolding*, relying on a sequence-based RNA representation as input.

Deep docking aims to predict the biomolecule-ligand pose, taking as input separate 3D structure-based representations of the biomolecule (or a pocket of it) and of the ligand. It can either be framed as a regression task, in which a unique pose is predicted from a ligand-target pair, or as a generative task, where a pose generative model is trained conditioned on a ligand-target pair. Several deep docking models have been proposed for protein-small molecule docking, including EquiBind^13^ and DiffDock.^14^ The first similar model for RNA-small molecule docking, NucleoDock,^71^ tackles RNA structural data scarcity by pretraining on structures generated by physics-based docking tools and fine-tuning on experimental structures. It outperforms several physics-based docking approaches including rDock and NLDock on the PoseBusters benchmark,^72^ showcasing the potential of deep learning approaches for RNA-ligand docking.

Co-folding aims to predict the RNA-ligand pose from RNA sequence and ligand Simplified Molecular Input Line Entry System (SMILES)^73^ representations (encoding the 2D chemical structure of the ligand). Therefore, despite requiring 3D structural supervision during training, a cofolding model enables prediction of RNA-small molecule poses without prior knowledge of their 3D structure. It can also potentially capture the specific conformational changes induced upon ligand binding, which is valuable in RNA-targeting drug design given the flexible nature of RNAs.

This has led to the development of several recent cofolding models, trained on diverse biomolecules (proteins, RNAs, DNAs) and their complexes, including biomolecule-biomolecule and biomolecule-ligand interactions. RoseTTAFold All-Atom,^74^ AlphaFold3,^75^ Chai-1,^76^ and Boltz-1 ^77^ can all perform RNA-small molecule cofolding using generative approaches such as diffusion models.

Despite these advances, the inherent flexibility of RNAs that makes cofolding valuable also complicates the prediction task. Indeed, RNAs can adopt multiple energetically favorable conformations,^78^ making it challenging to predict the actual 3D structure of the RNA-ligand complex under consideration. Moreover, cofolding remains computationally expensive, ranging from 5.9 GPU minutes to 1.5 GPU hours for models such as AlphaFold3,^75^ though some, such as Boltz-2,^79^ achieve faster prediction times (20 GPU seconds). Additionally, most models require multiple sequence alignments (MSAs) of RNAs as inputs, which cannot be computed for all RNAs, especially orphan RNAs, and are computationally expensive to generate. Finally, the performance of cofolding models critically depends on the quality of MSA inputs.^80^

#### 2.4.2 Pose Scoring

In *pose scoring*, a machine learning model is trained to predict binding propensity from docking poses. This task takes an RNA-ligand complex *C* as input and outputs a score *y* assessing binding propensity. The trained model can then be used as a learned scoring function that replaces the scores based on physical force fields used in classical docking approaches.

Early statistics-based RNA-small molecule scoring functions, including LigandRNA,^81^ DrugScoreRNA,^82^ and SPA-LN,^83^ relied on statistical potentials fitted to experimentally determined poses using inverse Boltzmann relation. These were followed by machine learning approaches, primarily relying on traditional machine learning models using interaction fingerprints.^84–88^ The only structure-based machine learning approach to RNA-small molecule pose scoring was proposed by RLaffinity.^89^ Although it displays competitive performance metrics in binding affinity prediction, the absence of redundancy removal in the data set and the reliance on random splitting make it unclear whether these scores reveal generalization ability and physical validity or mere memorization of the training examples (see Section 4.2 for further discussion).

The combination of established physics-based docking programs with machine learning-based scoring functions offers the potential to merge docking accuracy with machine learning’s ability to capture complex dependencies that exceeds previous scoring functions. However, this approach remains computationally expensive and time-consuming as the pose generation requires approximately 1 min per compound using docking.

Co-folding models can be trained jointly with a pose scoring model, showing promising capabilities in virtual screening. AlphaRank,^90^ by fine-tuning the cofolding model Protenix^91^ for protein binding, outperformed specialized models on the JACS 8 ^92^ lead optimization data set in a continuous binding affinity prediction setting. Boltz-2 ^79^ inherently includes binding affinity prediction in addition to cofolding and also outperforms specialized models on protein–ligand benchmarks, both in a virtual screening setting on a subset of the MF-PCBA data set^93^ and in a hit-to-lead and lead optimization setting on a subset of the FEP+ benchmark.^94^ However, its performance has not been assessed on RNA. We propose a first evaluation of its performance in RNA-ligand binding affinity prediction in Section 5.2.

### 2.5 Direct Scoring

Small molecule scoring can also be performed directly, without inferring the pose as an intermediary step: this consists of direct scoring, which can itself be formulated as three distinct tasks given the nature of its inputs.

#### 2.5.1 Quantitative Structure–Activity Relationship (QSAR)

The direct scoring of small molecules can be implemented through *Quantitative Structure–Activity Relationship* (QSAR) modeling, a widely used method in practice.^95^ QSAR formulates direct scoring as a machine learning task where the only input is a small molecule *L*. It is a purely ligand-based optimization technique that does not require access to RNA structures or features. It abstracts the mechanistic understanding of RNA-ligand interactions, but relies on prior knowledge about ligands for the RNA of interest.

Depending on the ligand training database, this approach can be used to predict the ability of a compound to bind RNAs in general,^96^ within specific RNA families,^97,98^ or for individual RNAs.^99–101^

Nevertheless, QSAR displays limitations. Abstracting from the drug-target interaction complicates scaffold hopping and the modeling of activity cliffs.^102^ Thanks to their ability to capture nonlinear relationships, QSAR models relying on neural networks with expert-based^98,99^ or structural^101,103^ features as input, might be more robust than linear QSAR models^100^ in these difficult but important cases.

Moreover, training QSAR models requires prior knowledge of many binding affinities of small molecules for the target RNA. This makes such models valuable in late stages of hit identification or for the hit-to-lead and lead optimization phases, when experimental affinity data have already been collected for the target RNA, but limits their applicability at early stages of hit identification, when only a small number of known binders are available for training. In this context, incorporating explicit information about the target, thus allowing transfer of knowledge acquired on various RNA targets to the target of interest, is essential.

#### 2.5.2 Binding Affinity Prediction

To this end, *binding affinity prediction* methods incorporate both an RNA *R* and a small molecule *L* as independent inputs (unlike pose scoring methods) and output a binding score *y*. The most accurate computational approaches rely on free energy perturbations. These 3D methods leverage molecular dynamics simulations to compute free energy differences between bound and unbound states. However, these approaches have a high computational cost. Machine learning has emerged as an alternative to accelerate binding affinity prediction.

Early models used expert-based RNA features encoding sequence,^104^ secondary structure,^105^ or function,^106^ generally focusing on specific RNA families, such as microRNAs.^105,106^ Following successes in protein modeling,^9,10,107^ the field transitioned toward structure-based geometric deep learning models, such as RNAmigos 2 ^108^ and GerNA-Bind,^36^ which are currently among the top-performing approaches. This shift toward deep learning also marked a transition from family specific to family agnostic methods. Recent methods increasingly leverage multimodal models combining sequence and structure.^37,109–112^

In addition to RNA representation, models differ by their RNA input *R*. While some process whole RNAs (e.g., RNAsmol^113^), others focus on an RNA pocket (e.g., RNAmigos2 ^108^). Building on the observation that many RNA binding sites are located near loops,^41^ ZHMol-RLinter^114^ and DistRMI^115^ specifically take loop motifs as input.

Finally, different encoders can be used for the small molecule *L*, with two major evolutions. First, the MACCS fingerprints^116^ that were commonly used^104,106^ were replaced with graph or SMILES^73^ representations (e.g.,^109^). Second, the adoption of ligand foundation models has become a dominant paradigm, following.^108,117^ These foundation models generally fall into three categories, based on their input representation: graph-based (e.g., Mole-BERT,^118^ adopted by^34,119^ ), SMILES-based (e.g., ChemBERTa,^120^ used in^111,121^ ), or multimodal (e.g., MolMVC,^122^ utilized by^110^). An exhaustive classification is provided in Table S2.

Despite this architectural progress, Volkov et al.^123^ uncovered a critical limitation regarding binding affinity prediction models: they tend to learn binding affinity from the small molecule alone (effectively behaving as QSAR models), rather than capturing target-ligand complementarity. This behavior limits their applicability and favors nonspecific binders. This risk also theoretically extends to pose scoring models, since they could extract ligand information from the pose. However, these models were empirically shown to be less prone to ignoring the target.^123^

To quantify this behavior, we propose a permutation experiment in Section 5.2.2. Preventing this behavior requires carefully designing and splitting the data sets (Sections 4.2 and 5.1). In the case of binary affinity prediction, we recommend that negative examples involve all RNAs and all ligands that appear in positive pairs.

#### 2.5.3 Target-Aware Ligand Prediction

Direct scoring can alternatively be formulated as a *ligand prediction* task, which takes an RNA *R* as input and outputs a small molecule *L* likely to bind the RNA *R*. Early ligand prediction approaches include InfoRNA^124^ and RNALigands,^125^ which mine RNA motifs and query databases of known interactions between RNA secondary structure motifs and small molecules. These were followed by deep learning approaches, RNAmigos^126^ and E3NN,^127^ which showed similar performance on ligand MACCS fingerprint reconstruction (i.e., recovering the correct ligand fingerprint).

Similar to binding affinity prediction approaches, ligand prediction approaches can be applied to targets lacking known binders. However, ligand prediction models typically generate only a single ligand per target, whereas the distribution of the possible ligands might be multimodal. This limitation impedes comprehensive exploration of chemical space and fails to capture the diversity of candidate binding modes and ligands for a given target.

To address this limitation, conditioning small-molecule *de novo* design generative models on structural information about the target represents a promising direction that could better account for the existence of multiple chemically distinct ligands that may bind the same target. While target-conditioned small molecule *de novo* design gained significant attention in protein research,^128–131^ it remains unexplored for RNA targets.

### 2.6 RNA Foundation Models

While models designed for specific tasks require annotated data (e.g., labeled images in computer vision, or affinity values in the case of RNA affinity prediction), foundation models have emerged as a powerful paradigm to leverage unlabeled data which is often available in much larger amounts. Foundation models are *pretrained* on this unannotated data, on a task defined solely from the data: typically predicting a masked part of the input data from its context. By solving this pretext task, they learn meaningful, general-purpose representations of the samples, which can then be fed to specialized models.

Foundation models have become the gold standard in computer vision, with models such as Dino,^132^ as well as in natural language processing where they are also known as Large Language Models (LLMs). In biological research, several foundation models have emerged for protein, DNA, and RNA sequences, also called protein/DNA/RNA language models.^58^ RNA language models include RNABert,^133^ RNA-FM^134^ and others.^135–137^ All are pretrained by masked language modeling, i.e., predicting the identity of masked nucleotides from their context. Some models introduce additional pretraining objectives: MP-RNA^138^ and StructRFM^137^ also predict masked secondary structures, RNABert follows a contrastive objective (learning similar embeddings for positions aligned in Rfam^139^), and RNAErnie^140^ also predicts structural motifs.

RNA language model embeddings are widely used for binding affinity prediction and binding site prediction tasks, sometimes as the sole RNA input representation, most often in combination with other RNA representations. Structural foundation models are still in their infancy. However, a step in this direction has come with the recent Ribosphere^141^ model, proposing the first structural generative pretraining: a model is trained to produce discrete embeddings of nucleotides enabling an accurate reconstruction of the global RNA backbone structure. The learned embeddings demonstrate interesting performance in RNA-ligand binding affinity prediction. RNAincoder^142^ can be considered as an expert feature-based foundation model: it encodes RNAs and their interaction partners (proteins, small molecules) using autoencoders operating on physicochemical, sequence-based and structural expert-based features.

## 3 RNA Encodings

We defined six machine learning tasks supporting drug discovery, as presented in Figure 2. Each of these tasks (except QSAR) involves RNAs as inputs or outputs. However, biological objects such as RNAs are not inherently machine-understandable. Therefore, an *encoding* is required to map biological objects to machine-readable vectors. The choice of the encoding represents a core part of machine learning model design, since its relevance to the specific task is critical for capturing meaningful information from the data and achieving high prediction performance. Encoding involves two steps. First, we map each object to a mathematical *representation* (e.g., a scalar, a vector, a graph, or text). Second, we apply a relevant machine learning model (referred to as an *encoder*) to this representation. For instance, if the selected representation is the nucleotide sequence, the encoder must be selected from models tailored for sequence or text data, such as a Transformer or a 1D Convolutional neural network (CNN). The encoder determines the types of mappings that can be learned between the chosen representation and the final vector.

In the context of RNA-targeting drug design, the biological objects to encode are small molecules, RNAs, and RNA-small molecule complexes. The encoding of small molecules has been extensively studied in chemoinformatics, including in machine learning contexts for drug discovery, with recent reviews available.^143,144^ Since these molecular representations are readily applicable to RNA drug design, we focus in this section on RNA encodings.

The multilevel nature of RNA structure (primary, secondary, tertiary, quaternary) offers a rich landscape of mathematical modeling possibilities. We first detail RNA sequence-based mathematical representations and their associated machine learning models, then we present RNA structure-based representations and their associated machine learning models.

Figure 3 provides an extensive mapping of different machine learning methods based on the representations they rely on. We note that some representations are not compatible with all tasks; for instance, sequence representation is not applicable to pose scoring, which requires input information regarding the 3D pose. Table A.2 in the Appendix provides a comprehensive overview of all reviewed methods and their encoding choices for RNAs, small molecules, and RNA-small molecule complexes.

### 3.1 Sequence-Based Encodings

RNAs can be represented by their nucleotide sequence, also known as their primary structure.

#### 3.1.1 Expert-Based Descriptors

The most straightforward approach for encoding RNA sequence relies on a vector of expert-based descriptors. They are often combined with structure-based expert-based features in a single vector, which can be directly used as input to shallow machine learning models such as random forests, support vector machines (SVMs), or linear and logistic regressions.

These features typically include RNA length and frequency of nucleotide k-mers (substrings of length k of RNA sequences). In addition to k-mers, some methods propose to predict the secondary structure then extract features such as the number of base pairs or secondary structure elements (bulges, hairpins) from this predicted secondary structure. For instance, SMTRS^104^ predicts the secondary structure using the ViennaRNA package^145^ then computes secondary structure-based features and RSAPred^146^ extracts features using RepRNA server,^147^ which itself relies on secondary structure prediction to compute such features. Sequence-based features might also include features encoding the evolutionary conservation of nucleotides from multiple sequence alignment (MSA). For instance, CapBind^48^ computes a feature quantifying the evolutionary conservation at each nucleotide position in the RNA sequence using MSA data.

The vast majority of machine learning methods for RNA-targeting drug design rely on expert-based features calculated from the RNA sequence. Such methods draw on solid domain knowledge and are highly interpretable and computationally efficient. However, they represent RNAs with fixed, predefined features which may fail to capture the full complexity of RNA and RNA-ligand interactions.

#### 3.1.2 Raw Sequence Representations

An alternative approach represents RNA based on the one-letter base code of its raw sequence of nucleotides. This sequence, encoded as textual data, can then be fed to machine learning encoders with architectures adapted from natural language processing. Such machine learning models are expected to learn longer range dependencies than the ones captured by models relying on expert-based features such as k-mer frequencies.

This approach has the advantage of being amenable to systems with no solved 3D structures, and hence to rely on large-scale data sets. This led to a proliferation of RNA language models in recent years.^133–135,140,148,149^ Such generalist models can encode RNA sequences into embeddings (vector representations), which can subsequently be used for drug design applications. For instance, the specialized models DeepRSMA^109^ and GerNA-Bind^36^ rely on the generalist RNA language model RNA-FM^134^ to encode RNA sequences.

### 3.2 Structure-Based Encodings

Recognizing that RNA binding properties are strongly dependent on structural characteristics,^1,20^ several approaches explicitly incorporate structural geometry. In protein research, structure-based approaches outperform state-of-the-art sequence-based models on several machine learning tasks relevant to drug design,^150^ providing grounds for optimism regarding similar RNA representations.

#### 3.2.1 Expert-Based Descriptors

As for sequence (Section 3.1.1), the most straightforward encoding of RNA secondary or tertiary structure is a set of expert-based descriptors, most often combined with sequence-based expert features.

Structural expert-based features generally include geometric descriptors of nucleotides, for instance the Laplacian norms of nucleotide coordinates in RNASite^42^ and ZHMolReSTaSite.^151^ They might also include features describing the size, shape and polarity of accessible surface areas, which are the RNA surfaces accessible to the solvent. For instance, DrugPredRNA^44^ relies on FreeSASA^152^ to compute such features. In addition, some methods propose to construct graphs whose nodes represent RNA nucleotides and edges represent noncovalent interactions and compute features encoding the graph topology. For instance, CapBind^48^ computes such a graph and uses the degree and closeness of the nucleotide nodes as features.

Some approaches model the RNA-ligand complex rather than RNA alone. In such cases, the feature vectors must capture interaction-specific properties. Relevant complex descriptors include geometric features encoding the relative positioning of RNA and ligand atoms,^85,86,88^ docking scores,^85^ and chemical fingerprints.^84^

#### 3.2.2 Full 3D Representations

To tackle complex structural dependencies, alternative approaches represent RNA structure in its full 3D form. Such representations can be combined with geometric deep learning^153^ (generalization of deep learning to non-Euclidean data, such as 3D shapes and graphs) encoders, thus enabling the modeling of complex relationships. 3D representations are essential for intrinsically geometric tasks such as docking or pose scoring, and can provide valuable geometric information for other drug design applications. These representations encompass various mathematical formulations, including multiview images, 3D grids, 3D graphs, and surface meshes.

Voxel grids generalize 2D images to 3D space, representing structures as sets of voxels (3D pixels). BiteNet^55^ adopts this approach for RNA structures, whereas RLaffinity^89^ applies it to RNA-ligand complexes. Grids enable accurate 3D modeling and leverage well-established convolutional neural networks as encoders. However, this approach is computationally inefficient since the molecular structure occupies only a small fraction of the 3D grid, leading to substantial computational waste.

All-atom 3D graphs model RNA structure as graphs where nodes represent heavy atoms and edges represent covalent bonds. These can be combined with E(3)-equivariant graph neural networks (EGNNs),^154^ which take into account atomic coordinates during learning, as proposed in E3NN^127^ or in the Geometric Vector Perceptron (GVP),^155^ introduced to learn on protein structures. For instance, ASRSMA^156^ leverages all-atom 3D graph RNA representations for RNA-small molecule binding affinity prediction and SMARTPocket^53^ relies on 3D graphs to identify binding sites.

Surface meshes represent RNAs as 3D surfaces discretized into triangles, each with distinct features. Building on the biological prior that biomolecular interactions are driven by surface geometry and chemistry rather than internal folds, this representation was initially developed for proteins in MaSIF,^157^ then adapted for RNAs in RLASIF^158^ for RNA binding site prediction and RLBSIF^54^ for affinity prediction.

However, full 3D representations present specific challenges: high dimensionality and reliance on 3D structural data, which are less abundant than sequence or secondary structure data, can lead to insufficient training data relative to model complexity. RNAs face this challenge even more acutely, as their structures are scarcer than protein structures. Using predicted 2D or 3D structures, as proposed in PRISM^112^ relying on Rhofold+^159^ to predict 3D structures, could overcome this issue. However, this approach introduces a risk of distribution shift relative to experimentally determined structures, as demonstrated for proteins by Huang et al.^160^ Moreover, current RNA structure prediction performance lags behind state-of-the-art protein structure prediction models,^161–163^ thereby limiting the performance of models relying on predicted RNA 3D structures.^164^

#### 3.2.3 Coarse-Grained Modeling

Coarse-grained modeling offers an alternative approach by abstracting fine structural details. This introduces a sparsity prior which is likely to reduce the computational complexity of models and tackle data scarcity.

Coarse-grained representations generally take the form of graphs whose nodes represent RNA residues. Edges can link adjacent residues in the backbone and residues involved in a base pair, as proposed in RNAmigos,^126^ or residues closer than a defined radius. Beyond node and edge definitions, RNA graphs can be customized by adding features to the graph nodes and edges or by distinguishing different edge types, which will be taken into account during the learning process.

The most straightforward approach uses 2D graphs that encode secondary structure. Their edges represent backbone connections and base pairs. For instance, GerNA-Bind^36^ relies on 2D graph modeling, among other representations. However, binding sites, especially in large RNAs, may involve nucleotides forming long-range, tertiary interactions and may therefore not be detectable by a representation leveraging secondary structure alone.^20^

2.5D graphs provide an intermediate level of abstraction: like 2D graphs, their edges encode backbone connections and base pairs, but they also include noncanonical edges. Moreover, these graphs possess different edge types accounting for the geometric families of base pairs, as defined by the nomenclature established by Leontis and Westhof.^165^ Therefore, this representation incorporates geometric information that is finer than raw base pairing but sparser than atomic coordinates. For example, RNAmigos,^126^ RNAmigos 2,^108^ and MultiModRLBP^49^ leverage 2.5D graph representations.

Finally, coarse-grained graphs may also be geometric (3D) graphs carrying 3D coordinates as node features. These coordinates could include either residue centroid positions, as proposed in RNABind,^50^ or the coordinates of a chosen set of representative atoms. For instance, GerNA-Bind^36^ uses 3D coordinates of C4’, N1, and P atoms as node features for each residue. In such graphs, edges are generally built based on the distances between nodes (either with a distance threshold or with k nearest neighbors).

#### 3.2.4 Modeling Conformational Ensembles

As sophisticated as they might be, the aforementioned structure-based RNA representations remain the representations of a single structure, whereas RNAs exist in an ensemble of interconverting conformations. This observation is particularly relevant to RNA-targeting drug design since the conformational ensembles of RNAs are redistributed upon ligand binding:^166^ small molecule binding can lead to the opening of cryptic binding sites which would not be easily detectable in the experimentally determined structure, and a small molecule without evident geometric complementarity with the reference structure of an RNA could bind one of its alternative or transient states.

Therefore, modeling this ensemble rather than a static structure would be a more accurate description of the RNA. A pioneering approach to accounting for RNA conformational flexibility in deep learning representations has been made in the inverse folding model gRNAde.^167^ The authors introduce a new representation called the multigraph: a higher-order generalization of a graph where an additional dimension represents the different alternative conformations of the RNA.

Instead of adding a dimension to the graph, Guo et al.^168^ add edges encoding the dynamics of residue pairs across conformations generated by molecular dynamics. Building on the finding that distograms (histograms of pairwise residue distances) predicted by folding algorithms also encode dynamics, we proposed to add edges and edge features from these distograms.^169^ We found this to outperform static representations of RNA on downstream tasks, including binding site prediction.

### 3.3 Multimodal Encodings

Several of these representations can be incorporated within a single multimodal model: a machine learning model that takes different data modalities as input and merges them within its architecture. In protein research, multimodal models such as AtomSurf^170^ or S3F^171^ proposed to combine sequence, graph and surface representations, outperforming state-of-the-art single-modality approaches.

Several multimodal approaches have been developed for RNA applications. Multimodal models for RNA-small molecule binding affinity prediction include MultiModRLBP,^49^ which integrates expert-based features, sequence, and 2.5D graph data; DeepRSMA^109^ and DeepMIF,^110^ combining sequence and 2D graph representations; and GerNA-Bind,^36^ which merges sequence, 2D graph, and 3D graph modalities. Outside drug design, HARMONY^172^ proposes a general-purpose multimodal model combining sequence, 2D and 3D graph representations.

The integration of embeddings from different modalities can be achieved through various strategies. Simple aggregation functions such as averaging or concatenation provide straightforward solutions, whereas cross-attention mechanisms, employed by DeepRSMA^109^ and RNAsmol,^113^ offer enhanced integration capabilities. Alternatively, some models incorporate RNA sequence language model embeddings as node features within graph representations, as demonstrated in RNAmigos2,^108^ DeepMIF,^110^ and RNABind.^50^

### 3.4 Comparing RNA Representations

When choosing a representation, several questions should be taken into consideration. Foremost is the representation’s expressiveness (i.e., the level of structural and spatial detail it encodes about the RNA). From this perspective, three-dimensional (3D) representations are inherently more expressive than two-dimensional (2D) representations, which in turn surpass sequence-based representations in their level of detail. However, this expressiveness should be balanced with the data availability. Indeed, the higher the dimension of the representation, the larger the training data set must be and the more computational power is needed.

RNA structures are far scarcer than RNA sequences (see Section 4.1). Using predicted 2D or 3D structures offers a solution, but this approach introduces distribution shifts relative to experimentally determined structures, as demonstrated by Huang et al. for proteins.^160^ Moreover, current RNA 3D structure prediction performance lags behind state-of-the-art protein structure prediction models,^161,162^ subsequently limiting the performance of models relying on predicted RNA 3D structures.^164^ RNA secondary structure and base pairing predictors^145,173–175^ are more accurate than existing 3D structure predictors, making the use of predicted structures a promising avenue for 2D and 2.5D representations specifically.

Empirical validation is thus needed to guide representation choices. Two benchmarks of RNA representations have been performed to date. The benchmark by Xu et al.,^164^ relying on predicted structures for structure-based encodings, found 2D graph representations to significantly outperform both sequence-based and 3D representations. Conversely, in our benchmark relying on experimentally determined structures,^176^ the 2D graph representation performed on par with the 3D graph representation, both still outperforming the sequence representation. This suggests that the performance of 3D graph representations on predicted structures was impeded by the inaccuracy of structure predictors. Moreover, we found the 2.5D representation to yield the best results, which might be attributed to the sparsity and biological relevance of this representation.

Beyond existing benchmarks, future work must deepen the experimental comparison of representations. This includes benchmarking across a wider array of tasks, especially those not intrinsically structure-related, and incorporating multimodal representations that combine sequence and structural information. These directions are essential for guiding optimal representation choices.

## 4 Data Sets and Splitting

Apart from the choice of representations and task formulation, the efficiency of machine learning models for RNA drug design relies heavily on the data sets they use for training and testing. Moreover, particular care is required to estimate the performance of learning-based methods, depending on the drug discovery task of interest. In this section, we discuss the current best practices and advocate for more rigorous model comparisons.

### 4.1 Data Sets

Training and testing machine learning models for RNA-targeting drug design may require access to three types of data: RNA sequence data, RNA structure data, and RNA-small molecule interaction data. In what follows, we detail the existing databases and tools providing such data, along with their strengths and limitations.

RNA sequence data can be retrieved from dedicated sequence databases such as Rfam^139^ (providing MSAs and family information), RNAcentral^177^ (focused on noncoding RNAs and containing over 35 million sequences), and the NCBI’s nonredundant nucleotide sequence database.^178^ The largest noncoding RNA sequence database system, RNAcentral,^177^ contains 35,400,760 RNA sequences in its 24th release (2025).

Beyond sequence data, structure information provides another crucial component for RNA analysis. Structural data, mostly extracted from the PDB, are far scarcer than protein structural data: as of January first, 2025, the PDB contained only 8,801 RNA structures, compared to 233,272 protein structures. This stark disparity is illustrated in Figure 4A, which compares RNA and protein structure counts in the PDB from 2000 to 2025. The data scarcity is even more pronounced when excluding rRNAs, which are highly structured through their interactions with proteins and are therefore easier to determine experimentally (see Figure 4C for a complete breakdown of PDB RNA structures by RNA categories).

**Figure 4:**
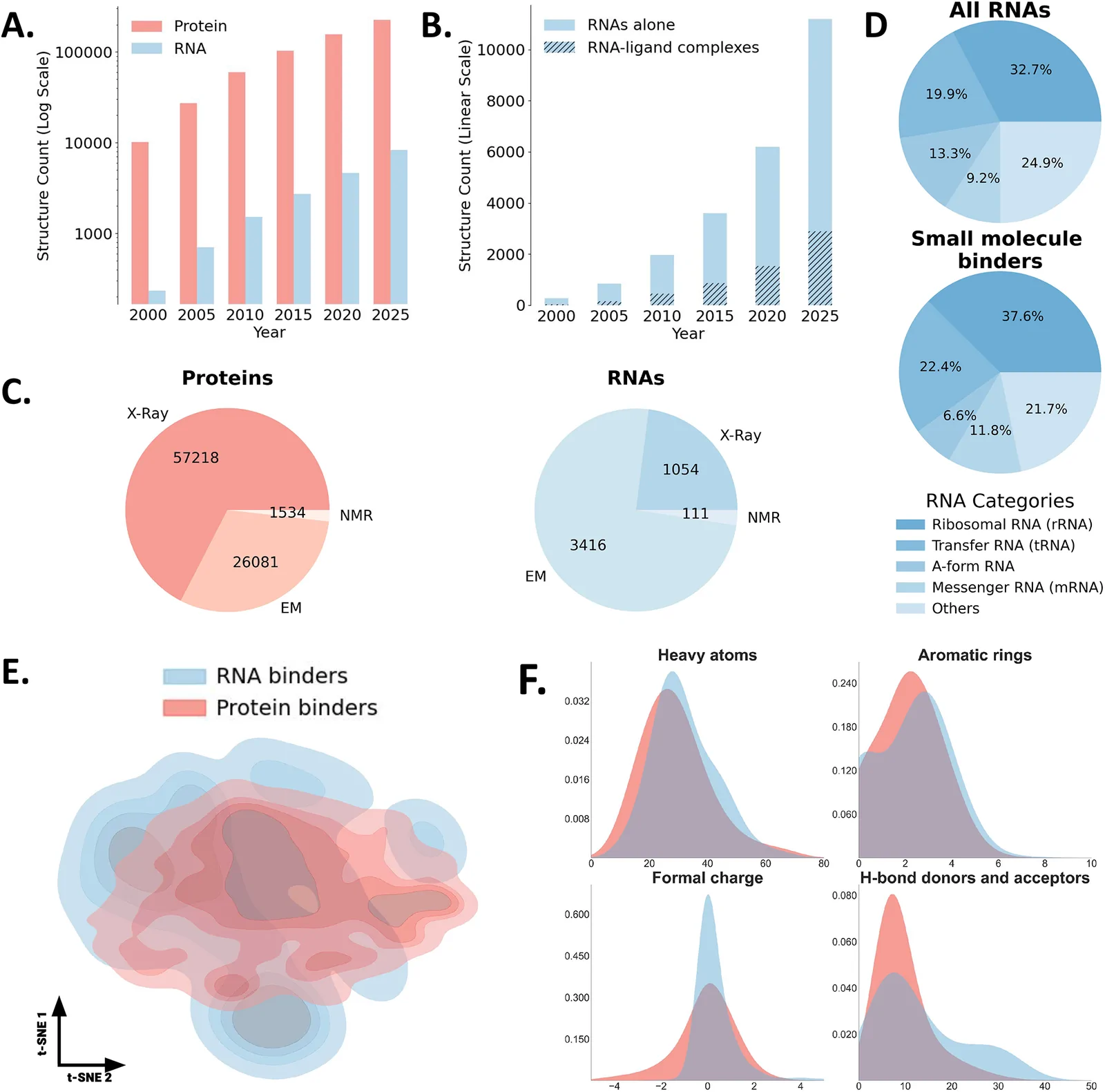
**A.** Evolution of the number of RNA and protein structures in the PDB. **B.** Evolution of the number of RNA structures in the PDB, both alone and ligand-bound. **C.** Distribution of experimentally determined protein and RNA structures (PDB entries) by primary technique (X-ray, Cryo-EM, NMR), released between 01/01/2020 and 11/27/2025. The specific time window was selected to control for time-dependent biases in the adoption rates of experimental methods. **D.** Repartition of RNA structures available in the PDB by category. **E.** t-SNE visualization of protein binders (in red) and RNA binders (in blue) in the chemical space. **F.** Distribution of chemical properties for proteins and RNA. Upper left: Number of heavy atoms. Upper right: Number of aromatic rings. Bottom left: Formal charge. Bottom right: Number of H-Bond donors and acceptors (total).

The scarcity of RNA structural data stems, in part, from experimental challenges specific to RNA structure determination: because of their higher flexibility, RNAs are harder to crystallize, thus limiting the use of X-ray crystallography. This could explain the smaller share of structures determined by X-ray among RNA structures compared to protein structures and, conversely, the larger share of structures determined by cryo-electron microscopy (cryo-EM), observed in Figure 4C.

RNA data require specific preprocessing. In particular, we recommend removing redundancy among RNA structures since the PDB structures contain several instances of the same RNA molecules. Specific tools facilitate the preprocessing and use of RNA structures from the PDB. For instance, BGSU^179^ removes redundancy among RNA structures, RNAsolo^180^ provides cleaned files in various formats through a web interface and RNANet^181^ links RNA structures with their corresponding MSAs.

When using RNA data for drug design, complementary data regarding RNA-small molecule interaction becomes necessary. Restricting to RNA-small molecule complex structures restrains the amount of data available, as illustrated by Figure 4B: as of January first, 2025, 3,076 RNA-small molecule complex structures were available, accounting for approximately 35% of all RNA structures. Several databases provide RNA data specifically curated for drug design applications. Some of them compile RNA-small molecule binding pairs alongside their binding affinities, RNA sequence and expert-based features characterizing small molecules, such as SM2miR^182^ (focusing on miRNAs; 2,925 interactions) and R-SIM^183^ (2,501 interactions). The protein-focused PDBbind database^184^ also offers 101 RNA-ligand pairs. Other databases provide experimentally determined RNA-small molecule complex structures, such as HARIBOSS^185^ (1,000 structures) and ROBIN.^96^

Drug design-specific preprocessing may encompass additional steps, such as removing protein-dominated complexes and selecting drug-like small molecules based on their mass or chemical properties.^56^ By comparing all RNA and protein binders contained in the PDBbind^184^ and R-SIM^183^ databases (Figure 4E and F), we observe that they follow different distributions in the chemical space. This underlines the need for machine learning models specific to RNA-targeting drug design, as well as the use of dedicated chemical libraries.

In particular, negatively charged compounds are disfavored, due to the high negative charge of RNA backbone. They include more hydrogen bond donors and acceptors, as they cannot rely on hydrophobic interactions for binding. They also tend to possess more aromatic rings than protein binders, which highlights the role of π-stacking in RNA-small molecule interactions. We propose an interactive Web site for a more extensive exploration of RNA- and protein-binder chemical spaces: https://wisskarrou.github.io/RNA_vs_protein_binders/.

### 4.2 Splitting

#### 4.2.1 Motivation

Once a data set has been selected and preprocessed, a key step for machine learning follows: data set splitting. Indeed, machine learning consists of three steps: model training (in which the model learns the input–output relationship), validation (first-pass evaluation of the model aiming to calibrate hyperparameters, which defines the type of input-output relations the model is allowed to learn) and test (final model evaluation aiming to give the users an idea of the confidence they can expect and comparing its performance with alternative models). If training, validation and test are done on the same data set, it is likely that the learned model will be specific to this data set: this phenomenon is called *overfitting*. Therefore, the performance of this model on external data sets will be much lower than the performance reported during the test phase. Since machine learning models are generally meant to be used on external data sets, such as a user’s proprietary data or new RNA targets being unveiled in our case, we aim to foster generalization ability and limit overfitting by splitting the data set into independent training, validation and test sets.

Splitting is generally achieved by randomly splitting the data set into three subsets, thus ensuring no data is duplicated between these sets. However, in structural biology and chemistry, this straightforward approach faces unique challenges. Indeed, two distinct RNA or protein structures might still be highly similar. For instance, they can correspond to a single macromolecule experimentally determined under different conditions or to evolutionarily close macromolecules. Similarly, distinct ligands can show great similarity when they belong to the same chemical scaffold. In other words, the independent and identically distributed (IID) data assumption is not always satisfied in structural biology and chemistry.^186,187^ Therefore, the choice of the splitting strategy is not straightforward: one might either want objects from the training, validation and test sets to be only distinct (weak generalization) or to enforce a dissimilarity level between them (strong generalization). The choice of the splitting strategy depends on the intended use of the model. Indeed, the performance metrics reported on the test set aim to reflect the expected model performance in real conditions. Therefore, the domain shift between training and test sets should mirror the shift expected between training set and real-world application data.

This shift depends on the stage of the drug design process and task performed. For instance, a binding affinity prediction model does not need the same generalization according to the context it is used in. Indeed, in early virtual screening, we generally have little data regarding the RNA and ligands of interest, therefore strong generalization is required for both RNAs and ligands. In hit-to-lead and lead optimization, data regarding the target RNA have already been collected during virtual screening. Therefore, a weak generalization might be sufficient for RNA, enabling the use of RNA random splitting, while strong generalization is still needed for ligands. Conversely, when repurposing a drug for a distinct target, the model should be able to generalize to new targets but not to new ligands. In these cases, weak generalization might be sufficient for ligands and strong generalization should be preferred for targets.^123^ Given these application-specific requirements, researchers have developed several splitting strategies.

#### 4.2.2 Similarity-Aware Splits

Enforcing RNA, protein or ligand strong generalization can be achieved through a similarity-based split: molecules are clustered according to their similarity and molecules belonging to the same cluster only appear in the training, validation or test set. Experiments by Li et al. (2017)^188^ and Yang et al. (2020)^189^ on proteins and Wyss et al. (2025) on RNAs^176^ demonstrated that random splitting leads to significantly inflated performance metrics compared to similarity-based splitting.

RNA similarity-based splitting could be defined either in terms of sequence- or structure-based similarity. For instance, RLBind,^46^ CapBind,^48^ and MultiModRLBP^49^ group RNAs based on sequence similarity using CD-Hit clustering.^190^ Other methods cluster RNAs based on their structural similarity, as measured by the TM-Score,^191^ used in RNABind,^50^ and the RMscore,^192^ used in DRLiPS.^193^

To enforce generalization across ligands, ligand similarity-based splitting clusters ligands using chemical similarity metrics. For instance, EMMPTNet^194^ and AnnapuRNA^86^ rely on the Tanimoto index. Another approach to ligand similarity-based splitting lies in scaffold splitting: grouping molecules based on the Murcko scaffold.^195^

In order to tune precisely the desired degree of generalization needed according to the expected similarity between training data and real conditions data sets, a recent approach named similarity aware evaluation^186^ can be leveraged. It proposes to build a test set with a user-defined distribution of molecules within each bin of similarity with the train set. This is particularly valuable in situations where the model is meant to be used on data sets showing both similar and dissimilar molecules to the ones of the training data set.

Since generalization across RNA families can be a specific challenge for deep learning models, RNA3DB data set,^196^ introduced for RNA structure prediction models, proposes a splitting strategy combining sequence-based, structure-based, and evolutionarily informed approaches. First, a sequence-based clustering is performed, then a graph encodes structural homology between the sequence clusters and Rfam^139^ families, and finally the graph is split into clusters, which can be used to form the train, validation and test sets.

#### 4.2.3 Time Split

As similarity-based split may be too strict and make the task excessively difficult compared to real conditions (especially in settings when the intended use of the model is on RNAs from the same family as those of the training set), an alternative approach to RNA or protein data splitting was proposed: time split.^197^

Time split^197^ amounts to splitting RNA or protein structures based on their release date (in the PDB for instance). It aims to replicate how data emerges during drug discovery, where models trained on previously determined structures are used to predict new ones, while avoiding training and testing on very similar structures that would have been released simultaneously in the PDB as part of the same experiment. Temporal splitting has been popularized by AlphaFold^198^ and widely used in protein pose generation^13,14^ as well as in structure prediction and cofolding.^75,79^ In RNA research, it is used by RNABind.^50^

#### 4.2.4 Recommendations

Many RNA-targeting drug design machine learning models still rely by default on random splitting. This may lead to inflated performance metrics and rewards memorization rather than genuine learning of the underlying physics. Instead, we advocate systematically discussing and grounding the splitting strategy choice.

The choice of the splitting strategy should be guided by the desired degree of generalization, which in turn depends on the intended use of the model in real conditions.

Alternatively, new models should be evaluated and compared with various splitting strategies, to provide performance estimates expected in various real-world scenarios, as proposed in GerNA-Bind.^36^

### 4.3 Benchmarks

In machine learning for RNA-targeting drug design, as often in machine learning, multiple computational models are developed to tackle the same tasks. Therefore, it is crucial to enable fair comparison between those models: testing them on the same data set, adopting the same splitting strategy and measuring the same evaluation metrics. We define a *benchmark* as the combination of a data set, a splitting strategy and a set of evaluation metrics.

Unfortunately, the majority of papers rely on their own data sets and splitting strategies, hindering fair comparison across models. This fragmentation significantly impedes the field by hindering the identification of the most promising modeling approaches, slowing overall progress, and potentially leading to redundant research efforts.

This fragmentation contrasts sharply with the more mature protein drug design field, which has successfully established several standard benchmarks. These include PDBbind,^184^ Davis,^199^ and KIBA^200^ for binding affinity prediction, CrossDocked,^201^ and PLINDER^202^ for protein–ligand docking tasks. These standardized benchmarks have enabled systematic progress in protein drug design by allowing researchers to build directly upon previous work and identify genuine advances.

Recognizing this critical gap in RNA research, recent efforts have begun developing similar standardized resources. In binding site prediction, the TR60 and TE18 data sets from RNASite^42^ have emerged as standards. Moreover, tools such as RNAglib^176^ aim to address this gap by providing standardized data sets, splitting strategies, and evaluation protocols across multiple RNA-related tasks, including binding site and binding affinity prediction. However, comprehensive benchmarks for sequence-based models remain an important area for future development.

## 5 Evaluation

In drug discovery, the adoption of a new model hinges on a convincing demonstration of its performance. While standard metrics are useful, they must be complemented by domain-specific measures that demonstrate real-world applicability. Ultimately, evaluation metrics should reflect how models will be used in practice to ensure meaningful comparisons and accurately assess their utility. Therefore, the choice of metrics is highly dependent on the task performed.

### 5.1 Evaluation Metrics

Binding site prediction is often framed as a node-level classification task, as the binding site is defined by the list of atoms or nucleotides. Therefore, the evaluation generally relies on standard classification metrics, such as Area under Receiver-operating curve (ROC-AUC), precision, or accuracy. Given the significant class imbalance between binding versus nonbinding sites, we emphasize metrics that account for imbalance, including Matthews correlation coefficient (MCC), F1-score, and balanced accuracy. Interestingly, RNACavityMiner^43^ validates its predicted binding sites by docking small molecules in it, and report docking metrics, assessing the practical use of their method in a drug design setting.

Pose generation tasks (deep docking or cofolding) must be assessed according to their ability to recover a structure close to the experimental structure. Models such as AlphaFold 3 ^75^ and Chai-1 ^76^ assess their cofolding performance through ligand RMSD: after aligning the protein or RNA atoms between structures, they compute the RMSD between predicted and experimental ligand pose.

When assessing pose scoring model capabilities, we must assess models’ ability to identify native-like conformations among a set of docking-generated poses. Therefore, a commonly used metric is the success rate: the success rate at X Å threshold denotes the percentage of RNA-ligand complexes whose best-scored pose (according to the pose scoring model) has a lower ligand RMSD than X Å to the native pose. This metric is for instance used in AnnapuRNA^86^ and RNAPosers.^85^

Finally, direct scoring methods (binding affinity prediction, ligand prediction or QSAR) can be assessed through standard classification or regression metrics, depending on the binary or continuous nature of their target variable.

Furthermore, these methods can be assessed using virtual screening metrics that better reflect practical applications. To compute such metrics, the models are tested on sets containing true ligands and decoys for each RNA. Then, compounds are ranked by predicted binding affinity. Success is measured by the model’s ability to rank native ligands higher than decoys. For instance, E3NN,^127^ RNAmigos2,^108^ and RNAsmol^113^ report the mean normalized rank of native compounds. RNAmigos2 additionally employs the X%-enrichment factor, denoting the factor by which the fraction of active ligands in the top-scoring X% of compounds exceeds the overall fraction of active ligands.

Given their closer alignment with practical applications, we advocate prioritizing metrics such as success rates and enrichment factors over metrics that lack direct operational meaning when developing machine learning models for RNA drug design.

To assess the performance of such virtual screening tasks, it is crucial to choose decoys whose distribution in the chemical space reflects the distribution of active compounds. Otherwise, models can learn to discard decoys based on their chemical difference to actives, overestimating model performances.^203^ Many efforts have been developed to select relevant decoys depending on the protein target at hand, for example through the DEKOIS^204^ or DUD-E^205^ databases. Chemical biases were also found for RNA negative compounds in the ROBIN data set. RNAdecoyDB proposes a data set of hard RNA decoys matching the chemical properties of active molecules.^103^

Beyond the ability to retrieve a large number of ligands, drug designers are interested in quantifying the diversity of retrieved compounds. Diverse hits provide more opportunities for hit optimization and reduce the risk of systematic failure during later stages in the drug design pipeline.^206^ The only paper investigating the diversity of its retrieved hits is RNAmigos2, which leverages visual checks and optimal transport to quantify the variety of compounds. We encourage future research to report metrics assessing the diversity of retrieved hits, including the number of unique scaffolds among the retrieved active compounds. For instance, ScaffAug^207^ proposes a metric named *SD*_100_ which quantifies the scaffold diversity among retrieved hits, along with data augmentation and reranking modules which can be plugged in to virtual screening models to enhance the diversity of their hits.

### 5.2 Evaluation of Binding Affinity Prediction Models

We conducted experiments to fill two gaps in the evaluation of binding affinity prediction models. First, the leading cofolding and binding affinity prediction model Boltz-2 ^79^ has only been assessed on proteins and not on RNAs, whereas it has been trained on RNA-small molecule affinity data. Second, the ability to learn specific interaction patterns rather than learning from ligand information alone is not evaluated by most existing RNA-small molecule binding affinity prediction models. The code and material necessary to reproduce the experiments are available at: https://github.com/wisskarrou/RNA_ligand_permutation_experiment.git

#### 5.2.1 Assessment of Boltz-2 Binding Affinity Prediction Capabilities on RNAs

The technical report for Boltz-2,^79^ a leading cofolding model for predicting binding affinity between proteins or RNAs and small molecules, reports an evaluation of its protein-small molecule prediction capabilities, which are outstanding, with an AuROC greater than 0.80 on the MF-PCBA benchmark.^93^ However, its RNA-small molecule binding affinity prediction capabilities were not assessed. Therefore, we assessed the binary binding prediction performance of Boltz-2 on the ROBIN data set^96^ to address this gap. We selected six RNA targets from ROBIN (namely SAM_ll, ZTP, TPP, PreQ1, NRAS, and RRE2B) and screened all ROBIN small molecules against them, reporting the exact same metrics as those reported for Boltz-2’s protein evaluation: the AuROC computed both at the global level (averaged across all RNA-small molecule pairs) and target level (average of the per-RNA averages) and the enrichment factors at 0.5, 1, 2, and 5% (Table 1).

**Table 1:** Performance of Boltz-2 on RNA-Small Molecule Interaction Prediction, Assessed on the ROBIN Database

	AuROC		Enrichment Factor			
	target	global	0.5%	1%	2%	5%
Boltz-2	0.530	0.535	1.068	1.063	0.988	1.180

Our experiment reveals that Boltz-2 performs poorly in RNA-small molecule binding affinity prediction, with near random metrics, far below the performance it reaches in protein-small molecule binding affinity prediction. This observation underlines the need for RNA-specific approaches to binding affinity prediction.

#### 5.2.2 Permutation Experiment to Measure Ligand Specificity

Regarding binding affinity prediction, Volkov et al.^123^ highlighted a tendency of deep learning models to predict binding affinity based solely on small molecule features (see Section 2.5.2). Similar results were recently obtained for RNA.^103^ However, standard performance metrics fail to reveal whether models capture meaningful RNA-small molecule interaction patterns or predict affinity from ligand features alone. Therefore, high performance metrics do not preclude the systematic selection of nonspecific binders, a particularly pronounced risk for RNAs (see Section 1).

Evaluation experiments designed to ensure models learn from macromolecule-ligand interaction patterns rather than ligand features alone have gained traction in protein research.^10,208–210^ In contrast, only RNAmigos 2 ^108^ has performed such validation for RNA-ligand systems. To address this gap, we conducted similar experiments on other open-source RNA-ligand binding affinity prediction models: Boltz-2,^79^ RSAPred,^146^ DeepRSMA,^109^ GerNA-Bind,^36^ and RNAsmol.^113^

As depicted in Figure 5, we propose an experimental protocol that systematically swaps RNA-ligand pairings in the test set by applying a permutation σ to the RNAs. Each triplet (*R*, *L*, *y*) where *R* is an RNA, *L* is a ligand, and *y* is the experimental binding label (propensity), is replaced by (σ­(*R*), *L*, *y*). We then compare model performance on this permuted data set to performance on the original data set. Unchanged performance despite permuted data suggests that the model relies primarily on ligand features, while decreased performance suggests that the model effectively captures specific RNA-ligand interaction information. This protocol has the advantage of being applicable without retraining models.

**Figure 5:**
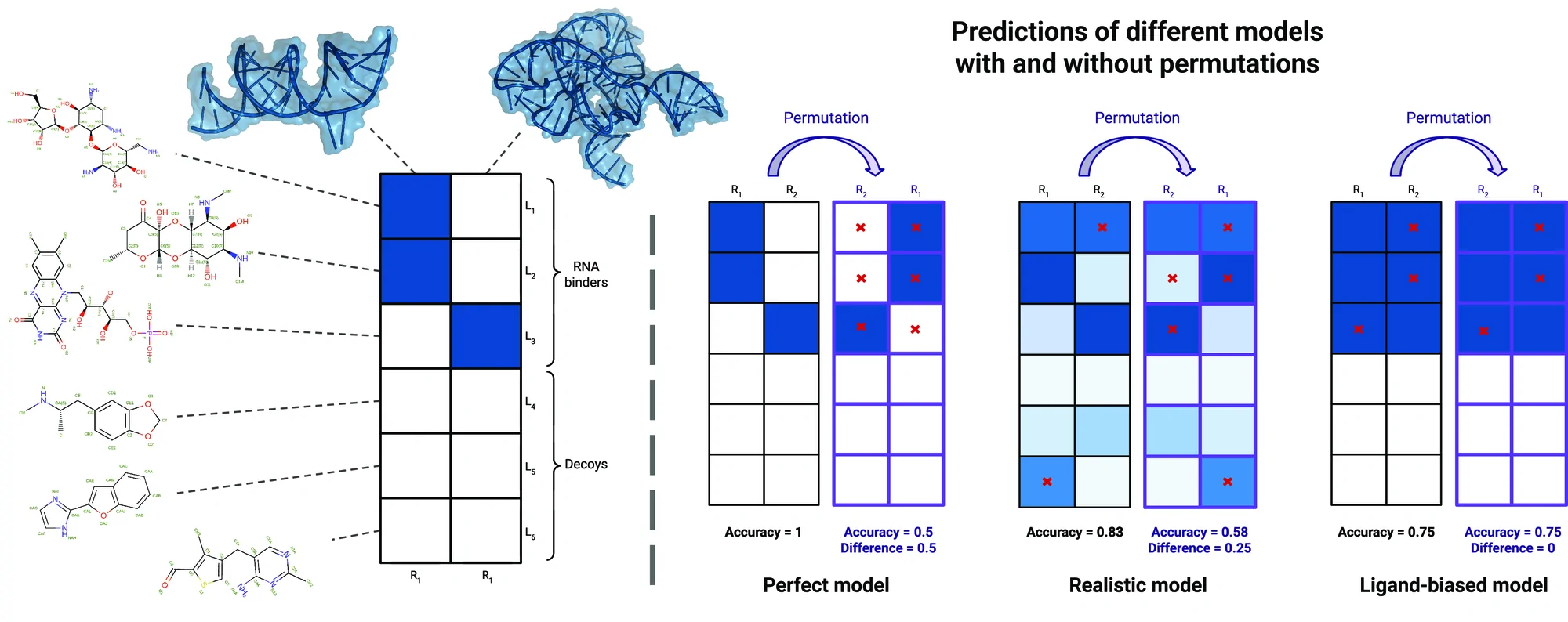
Impact of RNA permutation on model performance. A model that has captured genuine RNA-ligand affinity patterns will show a significant drop in performance when predictions are artificially swapped across different RNAs. In contrast, a ligand-biased model, relying solely on ligand features, will maintain consistent performance regardless of the RNA, as its predictions remain unchanged by the swap.

We present the results in Table 2. Metrics are reported in two ways: averaged across all RNA-ligand pairs (*global*), and averaged within each RNA target, then averaged across targets (*target*). A more detailed discussion of the setup and results of these experiments is provided in Section E of the Appendix.

**Table 2:** Results of the Swapping Experiments on RNA-Small Molecule Binding Affinity Prediction Models

	Metric		Without swapping	Target swapping	Performance gap (%)
Boltz 2	AuROC	global	0.535	0.533 ± 0.004	–0.4
		target	0.530	0.529 ± 0.002	–0.2
RNAmigos 2	AuROC	target	0.899	0.791 ± 0.007	–12.0
RSAPred	AuROC	global	0.600	0.670 ± 0.057	+11.7
		target	0.581	0.449 ± 0.047	–22.7
DeepRSMA	PCC	global	0.784	0.424 ± 0.064	–46.2
		target	0.430	0.351 ± 0.021	–22.5
GerNA-Bind	AuROC	global	0.783	0.514 ± 0.005	–34.4
		target	0.765	0.529 ± 0.005	–30.9
RNAsmol (Mol perturbation)	AuROC	global	0.974	0.975 ± 0.000	+0.1
		target	0.972	0.973 ± 0.002	+0.1
RNAsmol (Net perturbation)	AuROC	global	0.717	0.546 ± 0.009	–23.8
		target	0.651	0.569 ± 0.008	–12.6

As can be seen, performance significantly decrease for most models when the RNA targets are swapped, indicating specificity. This is particularly evident for RNAmigos 2,^108^ DeepRSMA,^109^ GerNA-Bind,^36^ and RNAsmol with network perturbations.^113^

However, methods such as RSAPred^146^ and RNAsmol with molecular perturbations^113^ show limited specificity, yielding nearly equivalent results before and after swapping. In the case of RNAsmol, this may be attributed to the data set construction. In the molecular perturbations setup, negatives are built by replacing the true ligand with a different molecule sharing similar MACCS properties. Hence, true ligands always appear as positives and the model can simply memorize them. Since training and testing sets share many small molecules as subsets of ROBIN, learning ROBIN ligands suffices to obtain high performance. This example illustrates a general pitfall in affinity prediction benchmarking: overly simplistic decoy construction can give rise to nonspecific, data set-dependent behaviors which inflate apparent performance.^103^

The limited performance and specificity of RSAPred^146^ may stem from its reliance on expert-crafted features, which may be insufficient to capture the geometric complementarity between RNA and ligand that structure-based models exploit. Indeed, RNAmigos 2,^108^ GerNA-Bind,^36^ and DeepRSMA^109^ all pass the permutation test, suggesting that structural reasoning is key to achieving specificity.

To provide a complete analysis, we also performed the symmetric experiment permuting ligands instead of RNAs, resulting in an even stronger performance drop. This indicates that models cannot predict scores from RNA features alone. This could be explained by the data distribution: the test set displays a stable proportion of active compounds per RNA target. Another explanation could lie in the higher expressiveness of ligand encodings, relying on more abundant data and a more mature field than their RNA counterparts, facilitating the direct prediction of the score from the ligand.

The outcome of this permutation experiment emphasizes the necessity to carefully design data sets and decoys. Indeed, when choosing decoys chemically different from RNA binders, the model will likely learn to predict whether a small molecule belongs to the RNA-binding region of the chemical space, overlooking the RNA target. Such models will display low RNA specificity and artificially inflated performance metrics. For future work, we recommend reporting the results of the proposed swapping experiment along with the unperturbed results.

Among existing methods, we recommend that experimentalists prioritize models that demonstrate both strong RNA-specificity in our experiments and good overall performance: DeepRSMA,^109^ GerNA-Bind,^36^ and RNAmigos 2.^108^

## 6 Discussion

In this review, we addressed four fundamental questions in machine learning for RNA-targeting drug design: how can machine learning contribute to the drug design pipeline, what are the possible machine learning methods tailored for RNA data, what data should be used and how models should be evaluated.

Machine learning can enhance RNA-targeting drug design at several key stages of the drug discovery pipeline: hit identification, hit-to-lead phase and lead optimization. Across this pipeline, existing models can detect binding sites, infer RNA-ligand poses (molecular docking), and predict whether they bind each other as well as their binding affinity. However, some topics widely addressed in protein machine learning remain unexplored for RNA, presenting compelling opportunities for further research. Following the recent NucleoDock,^71^ deep docking, widely used in protein research,^13,14^ could be further advanced for RNA. *De novo* ligand design through generative models conditioned on RNA targets or binding pockets could be translated from protein^129,130,211,212^ to RNA drug design. Such generative approaches offer superior chemical space exploration compared to conventional virtual screening by enabling the creation of novel molecular entities tailored to specific RNA targets. Nevertheless, ensuring synthetic accessibility of computationally generated compounds remains a critical consideration that must be integrated into model development.^213^

To implement these capabilities, machine learning models must rely on machine-understandable representations of RNA. Machine learning approaches to RNA-targeting drug design leveraged the multilevel structure of RNA to propose a wide range of mathematical representations such as raw sequence, 2D structure-based graphs or 3D graphs. However, current machine learning architectures and methodologies largely mirror those used in protein research, suggesting opportunities for RNA-specific innovations. In particular, accounting for RNA conformational flexibility in machine learning models represents a promising research avenue. While flexibility incorporation has already yielded modeling advances in RNA inverse folding^167,214^ and protein-targeting drug design,^215^ it remains unexplored in RNA-targeting drug design. The need for such methodologies will intensify if experimental advances, exemplified by techniques such as atomic force microscopy,^16^ ultimately enable high-throughput characterization of RNA conformational ensembles.

Beyond methodological considerations, the field faces significant data challenges. Indeed, RNA structures are far scarcer than their protein counterparts. Transfer learning from proteins to RNAs could thus help overcome the scarcity of RNA structural data. In transfer learning, a machine learning model initially trained on a domain with abundant data (e.g., protein structures) is subsequently specialized (*fine-tuned*) on a second data domain (e.g., RNA structures). Therefore, fewer data are required in the specialization domain since the model already benefits from the knowledge acquired on the first domain. Pioneering transfer learning approaches were proposed from proteins to RNAs for binding site prediction in RNet^56^ and SMARTPocket,^53^ and across several biomolecules in Atomica.^216^ Moreover, current models generally rely on specific preprocessing and data set splitting strategies, hindering fair comparison between models. Therefore, building standardized benchmark data sets for all tasks and ensuring their systematic adoption will be essential to field development.

Finally, appropriate evaluation remains crucial for meaningful progress. We advocate the use of application-oriented metrics adapted to the specific stage of the drug discovery pipeline where models are applied. We also emphasize the compelling need to assess and optimize binding affinity prediction models based on their capability to capture significant interaction patterns rather than raw binding affinity values and to retrieve diverse ligands. We hope the permutation experiment proposed in this review will advance this more nuanced evaluation approach.

Together, these considerations outline a clear path forward: leveraging generative modeling for new tasks such as deep docking and *de novo* design, developing RNA-specific methodologies that account for conformational flexibility, establishing standardized evaluation frameworks, and adopting more sophisticated metrics that align with drug discovery objectives. Addressing these challenges will be essential for realizing the full potential of machine learning in RNA-targeting therapeutics.

## Supplementary Material



## Notes

Details of the notations; comprehensive tables of RNA encodings (Table S1); small molecule encodings (Table S2); RNA–small molecule complex encodings (Table S3) for all reviewed machine learning methods, organized by drug design task; details on RNA–small molecule databases and their usage across reviewed methods; summary of data set splitting strategies employed by reviewed methods; methodology and PDB query parameters used to construct the data set figures, including chemical filtering criteria for drug-like small molecule selection; and Table S4: Full experimental setup and complete performance results of the permutation experiment assessing binding specificity of six open-source binding affinity prediction models (Boltz-2, RNAmigos 2, RSAPred, DeepRSMA, GerNA-Bind, and RNAsmol), including per-model results across multiple data sets and random seeds under RNA and ligand swapping conditions (PDF)

## Abbreviations

RNA: ribonucleic acid
ML: machine learning
MSA: multiple sequence alignment
QSAR: quantitative structure–activity relationship
