# Supplementary material for: Machine Learning for RNA-Targeting Drug Design
Source: J Chem Inf Model. 2026 Jul 14;66(14):7840–61. doi: 10.1021/acs.jcim.6c01094 (PMC13505966; doi:10.1021/acs.jcim.6c01094)
Supplement: Supplementary file 1 [file ci6c01094_si_001.pdf]

Supplementary Information:
Machine Learning for RNA-Targeting Drug Design

Wissam Karroucha[1,2,3], Carlos Oliver[4], Véronique Stoven[1,2,3], Vincent Mallet[1,2,3]*

[1]Mines Paris, PSL Research University, CBIO, Paris 75272, France
[2]Institut Curie, PSL Research University, Paris 75248, France
[3]INSERM, U1331, Paris 75654 France
[4]Vanderbilt University, Nashville, Tennessee 37235, US

*Email: vincent.mallet@minesparis.psl.eu

This Supplementary Information provides additional methodological details and extended results that complement the main text. **Section A** offers an exhaustive classification of the RNA–small molecule interaction prediction methods surveyed in this review, tabulating the RNA encodings, small molecule encodings, and complex encodings employed by each model. **Section B** describes the principal databases and datasets used across the reviewed studies, discussing their scope and the subset of methods that rely on each resource. **Section C** examines the data-splitting strategies adopted by the surveyed models, with particular attention to sequence-, structure-, and chemistry-based partitioning schemes and their implications for generalization assessment. **Section D** documents the data sources and construction procedures underlying each panel of the dataset characterization figure presented in the main text. Finally, **Section E** reports the full results of the perturbation experiment, providing per-model and per-dataset breakdowns under the target swapping and ligand swapping conditions.

A Extensive classification of methods

This section provides a comprehensive classification of the RNA–small molecule interaction prediction methods covered by this review. We characterize each method along three complementary axes: (i) how the RNA molecule is encoded (Section A.2), (ii) how the small molecule is encoded (Section A.3), and (iii) when applicable, how the RNA–small molecule complex as a whole is encoded (Section A.4). This three-way decomposition follows the unified notation introduced below and allows a structured comparison of architectural choices across tasks, ranging from binding site prediction to binding affinity estimation and ligand prediction.

A.1 Notations

In this section, we use the following notation: $\mathcal{R}^R$ denotes an RNA *representation*: a function mapping RNAs to mathematical objects. $\mathcal{E}_\theta^R$ denotes an RNA *encoder*: a machine learning model with parameters θ mapping these representations to vectors. $\psi_\theta^R = \mathcal{E}_\theta \circ \mathcal{R}$ denotes the complete *encoding*: the combination of the *representation* and *encoder*, thus mapping RNAs to vectors. The symmetric notations with L superscript apply to ligand representations, encoders and encodings.

A.2 RNA encodings

Table S1 catalogues the RNA encoding strategies used by each surveyed method. For every model, we record the structural level at which the RNA is described, the representation $\mathcal{R}^R$ used to convert that description into a mathematical object, and the encoder $\mathcal{E}_\theta^R$ that maps this representation to a fixed-dimensional vector. Grey cells indicate that no learnable encoder is applied to the corresponding representation (i.e. the representation is used directly as input to the prediction head). Methods that leverage multiple structural levels are listed on several rows.

Table S1: Summary of RNA encodings

		RNA encoding ψ_θ^R		
**Task**	**Model**	**Structure level**	**Representation** $\mathcal{R}^R$	**Encoder** $\mathcal{E}_\theta^R$
Binding site prediction	RNASite [1]	Structure	Expert-based features	
	RNACavityMiner [2]	Structure	Expert-based features	
	DrugPredRNA [3]	Structure	Expert-based features	
	NABS [4]	Structure	Expert-based features	
	BiteNet [5]	Structure	3D Grid	3D CNN
	RNet [6]	Structure	3D Grid	3D CNN
	RLBind [7]	Structure	Expert-based features	
	RNetsite [8]	Structure	Expert-based features	
	CapBind [9]	Structure	Expert-based features	
	MultimodRLBP [10]	Sequence	Sequence	RNABert [11]
		Structure	Expert-based features	1D CNN
		Structure	2.5D Nucleotides graph	RGCN [12]
	ZHmolReSTasite [13]	Structure	Expert-based features	
	RNABind [14]	Structure	3D Nucleotides graph	EGNN [15]
	DRLiPS [16]	Structure	Expert-based features	
	RLBSIF [17]	Structure	3D Surface	MoNET [18]
	RLsite [19]	Sequence	Sequence	ERNIE-RNA [20]
		Structure	2D Nucleotides graph	GAT [21]
	GATRsite [22]	Structure	2D Nucleotides graph	GAT [21]
	CoBRA [23]	Sequence	Sequence	ERNIE-RNA [20]
	SMARTPocket [24]	Structure	3D Atomic graph	Geometric Transformer [25]
Binding site and affinity prediction	DeepRNA-DTI [26]	Sequence	Sequence	RNA-FM [27] (pretrained)
	GerNA-Bind [28]	Sequence	Sequence	RNA-FM [27] (pretrained) + MLP
		Structure	2D Nucleotides graph	GAT [21]
		Structure	3D Atomic graph	Equiformer [29]
	SMARTBind [30]	Sequence	Sequence	RNA-FM [27] (pretrained)
	SMRTnet [31]	Sequence	Sequence	RNASwan-seq [31]
		Structure	Dot-bracket	CNN
Binding affinity prediction	RFSMMA [32]		Expert-based features	
	SMTRS [33]	Sequence	Expert-based features	
	SMAJL [34]	Sequence	Expert-based features	
	RNAincoder [35]	Sequence	Expert-based features	Stacked autoencoder [36]
	RSAPred [37]	Sequence	Expert-based features	
	RNAmigos2 [38]	Structure	2.5D Nucleotides graph	RGCN [12]
	RNAsmol [39]	Sequence	Sequence	1D CNN
	ZHMol-RLinter [40]	Structure	Expert-based features	
	BioLLMNet [41]	Sequence	Sequence	RNA-FM [27]
	DeepRSMA [42]	Sequence	Sequence	RNA-FM [27] (pretrained) + 1D CNN
		Structure	2D Nucleotides graph	GAT [21]

Table S1 (Continued)

Task	Model	RNA encoding ψ_θ^R Structure level	Representation $\mathcal{R}^R$	Encoder $\mathcal{E}_\theta^R$
	RLASIF [43]	Structure	3D Surface	MoNET [18]
	EMMPTNet [44]	Sequence	Expert-based features	
		Sequence	De Bruijn graph	GCN [45]
	DeepMIF [46]	Sequence	Expert-based features	MLP
		Sequence	Sequence	RNA-FM [27]
		Structure	2D Nucleotides graph	GAT [21]
	DistRMI [47]	Sequence	Sequence	1D Transformer
	ASRSMA [48]	Structure	3D Atomic graph	3D Transformer
	MCARSMA [49]	Sequence	Sequence	RNAErnie [50]
		Structure	2D Nucleotides graph	GAT [21]
	ncRD-LG [51]	Sequence	Sequence	BiRNA-BERT [52]
	PRISM [53]	Sequence	Sequence	1D CNN
		Structure	3D Nucleotides graph	EGNN [15]
Ligand prediction	RNAmigos [54]	Structure	2.5D Nucleotides graph	RGCN [12]
	E3NN [55]	Structure	3D Atomic graph	NequIP [56]
Deep docking	NucleoDock [57]	Sequence	Sequence	RNA-FM [27]
		Structure	2D Nucleotides graph	GCN [45]
		Structure	3D Atomic graph	Transformer

A.3 Small molecule encodings

Table S2 classifies small molecule encodings. We report the representation $\mathcal{R}^L$ used to encode the ligand and the corresponding encoder $\mathcal{E}_\theta^L$. As in Table S1, grey cells denote the absence of a learnable encoder.

Table S2: Summary of small molecule encodings

		Small molecule encoding ψ_θ^L	
**Task**	**Model**	**Representation** $\mathcal{R}^L$	**Encoder** $\mathcal{E}_\theta^L$
Binding affinity prediction	RFSMMA [32]	Expert-based features	
	SMTRS [33]	MACCS fingerprint	
	SMAJL [34]	MACCS fingerprint	
	RNAincoder [35]	Expert-based features	Stacked autoencoder [36]
	RSAPred [37]	Expert-based features	
	RNAmigos2 [38]	2D Atomic graph	OptiMol [58]
	RNAsmol [39]	2D Atomic graph	Graph diffusion convolution
	ZHMolRLinter [40]	MACCS fingerprint	
	BioLLMNet [41]	3D Atomic graph	Mole-BERT [59]
	DeepRNA-DTI [26]	3D Atomic graph	Mole-BERT [59]
	DeepRSMA [42]	SMILES sequence	Transformer
		2D Atomic graph	GCN [45]
	RLASIF [43]	3D Surface	MoNET [18]
	GerNA-Bind [28]	2D Atomic graph	GCN [45]
		3D Atomic graph	Equiformer [29]
	EMMPTNet [44]	Expert-based features	
		SMILES sequence	BiLSTM
	SMARTBind [30]	Expert-based features	
	SMRTnet [31]	SMILES sequence	MoLFormer [60]
		2D Atomic graph	GAT [21]
	DeepMIF [46]	SMILES sequence	MolMVC [61]
		2D Atomic graph	
	DistRMI [47]	2D Atomic graph	GCN [45]
	ASRSMA [48]	3D Atomic graph	3D Transformer
	MCARSMA [49]	SMILES sequence	ChemBERTa [62]
		2D Atomic graph	GCN [45]
	ncRD-LG [51]	SMILES sequence	ChemBERTa [62]
	PRISM [53]	SMILES sequence	1D CNN
		2D Atomic graph	GCN [45]
Ligand prediction	RNAmigos [54]	MACCS fingerprint	
	E3NN [55]	MACCS fingerprint	
		3D Atomic graph	Uni-Mol [63]
QSAR	sChemNet [64]	MACCS fingerprint	MLP
	Grimberg *et al.*, 2022 [65]	MACCS fingerprint	
		SMILES matrix	2D CNN
		Image	2D CNN
	Cai *et al.*, 2022 [66]	Expert-based features	
	Rizvi *et al.*, 2020 [67]	Expert-based features	
	Yazdani *et al.*, 2023 [68]	Expert-based features	
	Haga *et al.*, 2023 [69]	3D Atomic graph	GIN [70]
	TRN [71]	2D Atomic graph	GCN [45]

Table S2 (Continued)

Task	Model	Small molecule encoding ψ_θ^L Representation $\mathcal{R}^L$	Encoder $\mathcal{E}_\theta^L$
Deep docking	NucleoDock [57]	3D Atomic graph	Transformer

A.4 Complex encodings

A subset of methods, primarily those addressing pose scoring, encode the RNA–small molecule complex as a single object. Table S3 summarizes these complex encoding strategies, reporting the representation $\mathcal{R}^C$ and encoder $\mathcal{E}_\theta^C$ for each relevant model. These approaches typically operate on three-dimensional structural information about the bound complex, either through expert-crafted interaction features or through graph- and grid-based neural network architectures.

Table S3: Summary of complex encodings

Task	Model	Complex encoding ψ_θ^C Representation $\mathcal{R}^C$	Encoder $\mathcal{E}_\theta^C$
Pose scoring	SIFt-ML [72]	Expert-based features	
	RNAPosers [73]	Expert-based features	
	AnnapuRNA [74]	Expert-based features	
	ssMD [75]	Expert-based features	
	RmsdXNA [76]	Expert-based features	
	IRIS [77]	Expert-based features	
	AffiGrapher [78]	3D Atomic graph	Message passing neural network
	RLaffinity [79]	3D Grid	3D CNN

B Details about the datasets used

The availability and quality of curated RNA–small molecule interaction data is a key determinant of model development and benchmarking in this field. This section summarizes the principal databases drawn upon by the methods surveyed in this review, highlighting their scope, the type of interaction data they provide, and the subset of models that rely on each resource.

The Protein Data Bank (PDB) [80] is by far the most widely used data source, providing experimentally determined three-dimensional structures of RNA–ligand complexes. It underpins a broad range of methods including RNASite [1], RNACavityMiner [2], DrugPredRNA [3], NABS [4], DRLiPS [16], MultimodRLBP [10], RLBSIF [17], RNAmigos [54], RNAmigos 2 [38], DeepRNA-DTI [26], SIFt-ML [72], RNAPosers [73], AnnapuRNA [74], and RLAffinity [79]. It also serves as the primary source for the LigandRNA dataset [81], which is itself widely reused in the literature. The ROBIN database [68], which focuses on riboswitch–ligand interactions with measured binding affinities, is another frequently used resource, notably by RNAsmol [39], GerNA-Bind [28], and RNAmigos 2 [38]. E3NN [55], RNABind [14], and GerNA-Bind [28] additionally draw on HARIBOSS [82], a curated dataset of small molecule-binding RNA structures designed with particular attention to drug-likeness criteria. Two databases cater more specifically to feature-based approaches: SM2miR, which is specialized in miRNA interactions and is used by RFSMMA [32], SMAJL [34], and sChemNet [64]; and R-SIM [83], a collection of RNA–small molecule binding affinities used by RSAPred [37], DeepRSMA [42], BioLLMNet [41], and Boltz-2 [84].

C Details about the splitting strategies

The strategy used to partition data into training, validation, and test sets has a substantial impact on the difficulty of a benchmark and on how well reported performance figures reflect real-world generalization. Ideally, test molecules or targets should be sufficiently dissimilar from training examples to prevent information leakage. This section describes the splitting approaches adopted by the surveyed methods, which can broadly be grouped according to the similarity criterion used.

Sequence similarity-based clustering. Several methods partition the data by grouping RNA sequences with high mutual similarity, ensuring that closely related sequences do not appear in both training and test sets. This strategy is adopted by RNAPosers [73], RNASite [1], BiteNet [5], RLBind [7], CapBind [9], ZHMolReSTasite [13], RNet [8], MultimodRLBP [10], and DeepRNA-DTI [26].

Structural similarity-based clustering. An alternative approach uses structural rather than sequence similarity to define clusters, which may provide a more stringent separation for RNAs whose function is conserved across diverse sequences. RNAmigos 2 [38], RNABind [14], and DRLiPS [16] follow this strategy.

Chemical similarity-based clustering and temporal splitting. Some methods further partition data based on the chemical similarity between ligands, so that the test set contains structurally novel small molecules not represented in the training set. GerNA-Bind [28], sChemNet [64], AnnapuRNA [74], SPRank [85], and DeepRNA-DTI [26] use this criterion. In addition, GerNA-Bind [28] employs a temporal split for its binding site prediction task, where structures deposited after a given cut-off date constitute the test set, mimicking a prospective evaluation setting.

D Details about construction of the Figures

This section documents the data sources and processing steps used to generate each panel of the dataset characterization figure (Figure 4) presented in the main text, ensuring full reproducibility of the results shown.

Panels A–D (PDB-derived statistics). Figures 4A–D were constructed by querying the Protein Data Bank (PDB) [80]. For Figure 4A, RNA and protein structures were selected using the filters *Number of Distinct RNA Entities* > 0 and *Number of Distinct Protein Entities* > 0, respectively; data reported for each year correspond to the number of structures publicly available on January 1st of that year. Figure 4B retains RNA–ligand complex structures containing at least one RNA entity and at least one non-polymer entity with a molecular weight between 160 and 1,000 Da, a range broadly corresponding to drug-like small molecules. For the pie chart in Figure 4D, RNA structures were classified according to the functional categories defined by the Nucleic Acids Knowledgebase (NAKB) [86]; structures carrying multiple functional annotations were excluded from this panel.

Panels E and F (chemical space analysis). For Figures 4E and 4F, we assembled a compound set by combining RNA and protein binders from the PDBbind database [87] with RNA binders from the R-SIM database [83]. In both cases, we applied the drug-likeness filter proposed in HARIBOSS [82]: only molecules with a molecular weight between 160 and 1,000 Da, containing at least one carbon atom, and composed exclusively of the atom types C, H, N, O, Br, Cl, F, P, Si, B, S, and Se were retained. Figure 4E displays a t-SNE projection computed from the Morgan chemical fingerprints of the resulting compound set, providing a low-dimensional view of the chemical diversity spanned by RNA-targeting and protein-targeting ligands.

E Extensive results of the perturbation experiment

This section presents the full results of the perturbation experiment introduced in the main text. The experiment is designed to probe whether a model has genuinely learned to characterize RNA–ligand interactions,

or whether its predictions are primarily driven by either the RNA or the small molecule in isolation. To this end, we systematically evaluate each model under two perturbation conditions (target swapping and ligand swapping) and compare the resulting performance to that obtained on the original, unperturbed test sets.

Models and datasets

We benchmark six binding affinity prediction models for which open-source code is available: Boltz-2 [84], RNAmigos 2 [38], RSAPred [37], DeepRSMA [42], GerNA-Bind [28], and RNAsmol [39]. This selection spans a wide range of architectures and representation choices. RSAPred is a linear regression model operating on expert-crafted features. Boltz-2 is a sequence-based co-folding model not originally designed for RNA–ligand affinity prediction. RNAsmol encodes RNA sequences with a 1D CNN. RNAmigos 2 represents the RNA as a 2.5D graph processed by a graph neural network. DeepRSMA and GerNA-Bind rely on multimodal representations: DeepRSMA combines sequence and 2D graph encodings, while GerNA-Bind integrates sequence, 2D graph, and 3D graph encodings.

Inference is performed on the datasets originally used to evaluate each model, with one exception: Boltz-2, which was not evaluated on RNA–ligand data in its original publication, is here tested on a subset of the ROBIN dataset comprising all RNA–ligand pairs involving one of the following RNA targets: SAM_ll, ZTP, TPP, PreQ1, NRAS, and RRE2B. For RNAmigos 2, inference is performed on the ChEMBL decoy set. For RSAPred, results are reported for riboswitches from the ROBIN dataset. For DeepRSMA, inference relies on the five-fold cross-validation dataset extracted from R-SIM [83], as only the cross-validation model weights were fully available to us. GerNA-Bind is evaluated on both the Biosensor [88] and ROBIN [68] datasets; however, the only set of available model weights was trained on the full dataset, *including the test set*. Consequently, the reported results overestimate the model's expected performance in realistic deployment conditions; nevertheless, they remain informative for the purposes of this experiment, which concerns the origin of the learned signal rather than absolute predictive accuracy. Finally, RNAsmol is evaluated on two datasets (PDB and ROBIN), and separately for each of its three released variants: *RNA perturbation*, where negative examples are generated by pairing ligands with RNA sequences obtained by random shuffling of a known RNA's sequence; *Mol perturbation*, where negatives pair a known RNA with molecules drawn from a distinct chemical library yet exhibiting high MACCS fingerprint similarity to the original ligand; and *Net perturbation*, where negative labels are randomly assigned to uncharacterized RNA–small molecule pairs within the original dataset.

Experimental design

For each model and dataset, the perturbation experiment proceeds as follows. In the *target swapping* condition, the RNA partner of each test pair is replaced by a randomly selected RNA from the same test set, breaking the correspondence between RNAs and labels. In the *ligand swapping* condition, the small molecule partner is swapped analogously. To account for variability across random permutations, each swapping condition is repeated with three independent random seeds, and we report the mean and standard deviation of each metric across the three resulting datasets.

For each model we report the performance metrics used in the corresponding original publication. We additionally compute metrics at two complementary levels of aggregation: *global* metrics are computed across all RNA–small molecule pairs pooled together, while *target*-averaged metrics are computed by first evaluating each metric per RNA target and then averaging across targets. The target-averaged setting is particularly relevant for virtual screening applications, where a model is used to rank candidate ligands for a fixed RNA target. Note that target-averaged metrics are not reported under the ligand swapping condition (grey cells in Table S4): because ligands have been shuffled, the link between ligands and labels has been severed, making within-target ranking uninformative. Results for RNAmigos 2 are likewise reported only at the target level, as it is an inherently per-target ranking model.

Table S4 collects all numerical results.

Table S4: Results of the perturbation experiment carried out on binding affinity prediction models (RMSE: Root mean squared error; PCC: Pearson's correlation coefficient; SCC: Spearman's correlation coefficient; AuROC: Area under Receiver-Operating Curve; AuPRC: Area under Precision-Recall Curve)

	**Metric**		**Without swapping**	**Target swapping**	**Ligand swapping**
Boltz-2 [84]	AuROC	global	0.535	0.533 ± 0.004	0.506 ± 0.006
		target	0.530	0.529 ± 0.002	
	AuPRC	global	0.009	0.008 ± 0.000	0.008 ± 0.000
	Enrichment factor	0.5%	1.068	0.649 ± 0.247	
		1%	1.063	1.163 ± 0.023	
		2%	0.988	1.268 ± 0.078	
		5%	1.180	1.102 ± 0.058	
RNAmigos 2 [38] (PDB decoys)	AuROC	target	0.784	0.580 ± 0.020	
RNAmigos 2 [38] (Chembl decoys)	AuROC	target	0.899	0.791 ± 0.007	
RSAPred [37]	AuROC	global	0.600	0.670 ± 0.057	0.566 ± 0.002
		target	0.581	0.449 ± 0.047	
	AuPRC	target	0.352	0.457 ± 0.017	
	F1-Score	global	0.681	0.692 ± 0.039	0.651 ± 0.001
		target	0.455	0.474 ± 0.032	
DeepRSMA [42]	PCC	global	0.784	0.424 ± 0.064	0.256 ± 0.022
		target	0.430	0.351 ± 0.021	
	SCC	global	0.787	0.407 ± 0.082	0.255 ± 0.026
		target	0.405	0.333 ± 0.022	
	RMSE	global	0.895	1.414 ± 0.060	1.685 ± 0.088
		target	0.895	1.208 ± 0.095	
GerNA-Bind [28] (Biosensor)	AuROC	global	0.976	0.675 ± 0.010	0.632 ± 0.068
		target	0.967	0.728 ± 0.037	
	AuPRC	target	0.599	0.335 ± 0.028	
	F1-Score	global	0.715	0.266 ± 0.010	0.217 ± 0.024
		target	0.509	0.178 ± 0.003	
GerNA-Bind [28] (ROBIN)	AuROC	global	0.783	0.514 ± 0.005	0.557 ± 0.002
		target	0.765	0.529 ± 0.005	
	AuPRC	target	0.232	0.105 ± 0.003	
	F1-Score	global	0.264	0.128 ± 0.002	0.161 ± 0.003
		target	0.246	0.117 ± 0.002	
RNAsmol [39] (PDB) Mol perturbation	AuROC	global	0.983	0.986 ± 0.005	0.504 ± 0.052
		target	0.950	0.997 ± 0.003	
	AuPRC	target	0.886	0.789 ± 0.032	
	F1-Score	global	0.935	0.930 ± 0.014	0.543 ± 0.031
		target	0.857	0.761 ± 0.025	
RNAsmol [39] (PDB) RNA perturbation	AuROC	global	0.991	0.480 ± 0.038	0.990 ± 0.001
		target	0.000	0.354 ± 0.180	
	AuPRC	target	0.955	0.449 ± 0.179	
	F1-Score	global	0.962	0.441 ± 0.016	0.960 ± 0.010
		target	0.924	0.429 ± 0.127	
RNAsmol [39] (PDB) Net perturbation	AuROC	global	0.700	0.575 ± 0.052	0.539 ± 0.078
		target	0.723	0.615 ± 0.059	

Table S4 (Continued)

	Metric		Without swapping	Target swapping	Ligand swapping
	AuPRC	target	0.593	0.563 ± 0.028	
	F1-Score	global	0.649	0.518 ± 0.043	0.495 ± 0.083
		target	0.497	0.335 ± 0.023	
RNAsmol [39] (ROBIN) Mol perturbation	AuROC	global	0.974	0.975 ± 0.000	0.486 ± 0.018
		target	0.972	0.973 ± 0.002	
	AuPRC	target	0.968	0.974 ± 0.002	
	F1-Score	global	0.898	0.898 ± 0.002	0.546 ± 0.012
		target	0.889	0.897 ± 0.003	
RNAsmol [39] (ROBIN) RNA perturbation	AuROC	global	1.000	0.511 ± 0.024	1.000 ± 0.000
		target	0.500	0.506 ± 0.062	
	AuPRC	target	0.710	0.576 ± 0.040	
	F1-Score	global	0.999	0.521 ± 0.019	0.999 ± 0.000
		target	0.710	0.479 ± 0.014	
RNAsmol [39] (ROBIN) Net perturbation	AuROC	global	0.717	0.546 ± 0.009	0.604 ± 0.012
		target	0.651	0.569 ± 0.008	
	AuPRC	target	0.669	0.615 ± 0.003	
	F1-Score	global	0.062	0.038 ± 0.011	0.038 ± 0.006
		target	0.054	0.033 ± 0.009	

Supplementary References

(1) Su, H.; Peng, Z.; Yang, J. Recognition of small molecule–RNA binding sites using RNA sequence and structure. *Bioinformatics* **2021**, *37*, 36–42.

(2) Xie, J.; Frank, A. T. Mining for ligandable cavities in RNA. *ACS Med. Chem. Lett.* **2021**, *12*, 928–934.

(3) Rekand, I. H.; Brenk, R. DrugPred_RNA—a tool for structure-based druggability predictions for RNA binding sites. *J. Chem. Inf. Model.* **2021**, *61*, 4068–4081.

(4) Jiang, Z.; Xiao, S.-R.; Liu, R. Dissecting and predicting different types of binding sites in nucleic acids based on structural information. *Briefings Bioinf.* **2022**, *23*, bbab411.

(5) Kozlovskii, I.; Popov, P. Structure-based deep learning for binding site detection in nucleic acid macromolecules. *NAR Genomics Bioinf.* **2021**, *3*, lqab111.

(6) Möller, L.; Guerci, L.; Isert, C.; Atz, K.; Schneider, G. Translating from Proteins to Ribonucleic Acids for Ligand-binding Site Detection. *Mol. Inf.* **2022**, *41*, 2200059.

(7) Wang, K.; Zhou, R.; Wu, Y.; Li, M. RLBind: a deep learning method to predict RNA–ligand binding sites. *Briefings Bioinf.* **2023**, *24*, bbac486.

(8) Liu, H.; Jian, Y.; Hou, J.; Zeng, C.; Zhao, Y. RNet: a network strategy to predict RNA binding preferences. *Briefings Bioinf.* **2024**, *25*, bbad482.

(9) Xi, W.; Wang, R.; Wang, L.; Ye, X.; Liu, M.; Sakurai, T. An interpretable deep learning model predicts RNA–small molecule binding sites. *Future Gener. Comput. Syst.* **2024**, *159*, 557–566.

(10) Wang, J.; Quan, L.; Jin, Z.; Wu, H.; Ma, X.; Wang, X.; Xie, J.; Pan, D.; Chen, T.; Wu, T., et al. MultiModRLBP: A Deep Learning Approach for Multi-Modal RNA-Small Molecule Ligand Binding Sites Prediction. *IEEE J. Biomed. Health Inf.* **2024**.

(11) Akiyama, M.; Sakakibara, Y. Informative RNA base embedding for RNA structural alignment and clustering by deep representation learning. *NAR Genomics Bioinf.* **2022**, *4*, lqac012.

(12) Schlichtkrull, M.; Kipf, T. N.; Bloem, P.; Van Den Berg, R.; Titov, I.; Welling, M. In *The semantic web: 15th international conference, ESWC 2018, Heraklion, Crete, Greece, June 3–7, 2018, proceedings 15*, Springer: 2018, pp 593–607.

(13) Gao, J.; Liu, H.; Zhuo, C.; Zeng, C.; Zhao, Y. Predicting Small Molecule Binding Nucleotides in RNA Structures Using RNA Surface Topography. *J. Chem. Inf. Model.* **2024**, *64*, 6979–6992.

(14) Zhu, W.; Ding, X.; Shen, H.-B.; Pan, X. Identifying RNA-small molecule binding sites using geometric deep learning with language models. *J. Mol. Biol.* **2025**, 169010.

(15) Satorras, V. G.; Hoogeboom, E.; Welling, M. In *Proceedings of the 38th International Conference on Machine Learning*, ed. by Meila, M.; Zhang, T., PMLR: 2021; Vol. 139, pp 9323–9332.

(16) Krishnan, S. R.; Roy, A.; Wong, L.; Gromiha, M. M. DRLiPS: a novel method for prediction of druggable RNA-small molecule binding pockets using machine learning. *Nucleic Acids Res.* **2025**, *53*, gkaf239.

(17) Sang, C.; Shu, J.; Wang, K.; Xia, W.; Wang, Y.; Sun, T.; Xu, X. The prediction of RNA-small molecule binding sites in RNA structures based on geometric deep learning. *Int. J. Biol. Macromol.* **2025**, 143308.

(18) Monti, F.; Boscaini, D.; Masci, J.; Rodola, E.; Svoboda, J.; Bronstein, M. M. In *Proceedings of the IEEE conference on computer vision and pattern recognition*, 2017, pp 5115–5124.

(19) Sun, S.; Yang, J.; Gao, L.; Li, P.; Liu, Y. RNA language model and Graph attention network for RNA and small molecule binding sites prediction. *Bioinformatics* **2025**, btaf447.

(20) Yin, W.; Zhang, Z.; He, L.; Jiang, R.; Zhang, S.; Liu, G.; Zhang, X.; Qin, T.; Xie, Z. ERNIE-RNA: an RNA language model with structure-enhanced representations. *bioRxiv* **2024**, 2024–03.

(21) Veličković, P.; Cucurull, G.; Casanova, A.; Romero, A.; Lio, P.; Bengio, Y. Graph attention networks. *arXiv* **2017**.

(22) Sun, C.; Zhang, L.; Zhang, L.; Song, Y.; Ma, B.; Wang, Y. GATRsite: RNA–ligand binding site prediction using graph attention networks and pretrained RNA language models. *J. Chem. Inf. Model.* **2025**, *65*, 8448–8461.

(23) Jang, W.; Shin, W.-H. CoBRA: Compound Binding Site Prediction using RNA Language Model. *bioRxiv* **2025**, 2025–09.

(24) Thakare, R. H.; Taghavi, A.; Wang, J.; Childs-Disney, J. L.; Li, C.; Disney, M. D.; Li, Y. Geometric Deep Learning Reveals Ligandable and Cryptic RNA Binding Small Molecule Pockets (SMART-Pocket). *bioRxiv* **2026**.

(25) Krapp, L. F.; Abriata, L. A.; Cortés Rodriguez, F.; Dal Peraro, M. PeSTo: parameter-free geometric deep learning for accurate prediction of protein binding interfaces. *Nature communications* **2023**, *14*, 2175.

(26) Bae, H.; Nam, H. DeepRNA-DTI: a deep learning approach for RNA-compound interaction prediction with binding site interpretability. *J. Cheminform.* **2025**.

(27) Chen, J.; Hu, Z.; Sun, S.; Tan, Q.; Wang, Y.; Yu, Q.; Zong, L.; Hong, L.; Xiao, J.; Shen, T., et al. Interpretable RNA foundation model from unannotated data for highly accurate RNA structure and function predictions. *arXiv* **2022**.

(28) Xia, Y.; Li, J.; Chu, Y.-T.; Rao, J.; Chen, J.; Hua, C.; Yu, D.-J.; Chen, X.-C.; Zheng, S. Deciphering RNA–ligand binding specificity with GerNA-Bind. *Nat. Mach. Intell.* **2025**, 1–13.

(29) Liao, Y.-L.; Smidt, T. Equiformer: Equivariant graph attention transformer for 3d atomistic graphs. *arXiv* **2022**.

(30) Jiang, S.; Taghavi, A.; Wang, T.; Meyer, S. M.; Childs-Disney, J. L.; Li, C.; Disney, M. D.; Li, Y. Small Molecule Approach to RNA Targeting Binder Discovery (SMARTBind) Using Deep Learning Without Structural Input. *bioRxiv* **2025**, 2025–09.

(31) Fei, Y.; Wang, P.; Zhang, J.; Shan, X.; Cai, Z.; Ma, J.; Wang, Y.; Zhang, Q. C. Predicting small molecule–RNA interactions without RNA tertiary structures. *Nat. Biotechnol.* **2026**, 1–12.

(32) Wang, C.-C.; Chen, X.; Qu, J.; Sun, Y.-Z.; Li, J.-Q. RFSMMA: A New Computational Model to Identify and Prioritize Potential Small Molecule–MiRNA Associations. *J. Chem. Inf. Model.* **2019**, *59*, 1668–1679.

(33) Zhuo, Z.; Wan, Y.; Guan, D.; Ni, S.; Wang, L.; Zhang, Z.; Liu, J.; Liang, C.; Yu, Y.; Lu, A., et al. A loop-based and AGO-Incorporated virtual screening model targeting AGO-Mediated miRNA–mRNA interactions for drug discovery to rescue bone phenotype in genetically modified Mice. *Adv. Sci.* **2020**, *7*, 1903451.

(34) Shen, C.; Luo, J.; Lai, Z.; Ding, P. Multiview joint learning-based method for identifying small-molecule-associated MiRNAs by integrating pharmacological, genomics, and network knowledge. *J. Chem. Inf. Model.* **2020**, *60*, 4085–4097.

(35) Wang, Y.; Chen, Z.; Pan, Z.; Huang, S.; Liu, J.; Xia, W.; Zhang, H.; Zheng, M.; Li, H.; Hou, T., et al. RNAincoder: a deep learning-based encoder for RNA and RNA-associated interaction. *Nucleic Acids Res.* **2023**, *51*, W509–W519.

(36) Pan, X.; Fan, Y.-X.; Yan, J.; Shen, H.-B. IPMiner: hidden ncRNA-protein interaction sequential pattern mining with stacked autoencoder for accurate computational prediction. *BMC Genomics* **2016**, *17*, 582.

(37) Krishnan, S. R.; Roy, A.; Gromiha, M. M. Reliable method for predicting the binding affinity of RNA-small molecule interactions using machine learning. *Briefings Bioinf.* **2024**, *25*, bbae002.

(38) Carvajal-Patiño, J. G.; Mallet, V.; Becerra, D.; Niño Vasquez, L. F.; Oliver, C.; Waldispühl, J. RNAmigos2: accelerated structure-based RNA virtual screening with deep graph learning. *Nat. Commun.* **2025**, *16*, note: highlight, 2799.

(39) Ma, H.; Gao, L.; Jin, Y.; Ma, J.; Bai, Y.; Liu, X.; Bao, P.; Liu, K.; Xu, Z. Z.; Lu, Z. J. RNA–ligand interaction scoring via data perturbation and augmentation modeling. *Nat. Comput. Sci.* **2025**, *5*, 648–660.

(40) Zhuo, C.; Gao, J.; Li, A.; Liu, X.; Zhao, Y. A Machine Learning Method for RNA–Small Molecule Binding Preference Prediction. *J. Chem. Inf. Model.* **2024**, *64*, 7386–7397.

(41) Tahmid, M. T.; Abir, A. R.; Bayzid, M. S. BioLLMNet: Enhancing RNA-Interaction Prediction with a Specialized Cross-LLM Transformation Network. *bioRxiv* **2024**, 2024–10.

(42) Huang, Z.; Wang, Y.; Chen, S.; Tan, Y. S.; Deng, L.; Wu, M. DeepRSMA: a cross-fusion-based deep learning method for RNA–small molecule binding affinity prediction. *Bioinformatics* **2024**, *40*, btae678.

(43) Xia, W.; Shu, J.; Sang, C.; Wang, K.; Wang, Y.; Sun, T.; Xu, X. The prediction of RNA-small-molecule ligand binding affinity based on geometric deep learning. *Comput. Biol. Chem.* **2025**, 108367.

(44) Wu, Z.; Deng, Z.; Wu, Q.; Zuo, Y.; Pan, X.; Shen, H.; Fang, X.; Ge, Y.; Hu, S. Explainable RNA-Small Molecule Binding Affinity Prediction Based on Multiview Enhancement Learning. *J. Chem. Inf. Model.* **2025**.

(45) Kipf, T. N.; Welling, M. Semi-supervised classification with graph convolutional networks. *arXiv* **2016**.

(46) Song, J.; Gao, A.; Tian, S.; Yang, Q.; Deng, L.; Feng, Q.; Hou, M. DeepMIF: A Multiview Interactive Fusion-Based Deep Learning Method for RNA–Small Molecule Binding Affinity Prediction. *J. Chem. Inf. Model.* **2026**.

(47) Liu, Z.; Zhu, Q.; Tian, Q.; Zhong, L.; Deng, Y.; Wei, D.; Fu, H. DistRMI: a deep distance-aware neural network for explainable RNA loop motif-small molecule interaction prediction. *Briefings in Bioinformatics* **2025**, *26*, bbaf660.

(48) Li, Y.; Huang, Z.; Chen, S.; Wang, Y.; Wu, M.; Deng, L. In *2025 IEEE International Conference on Bioinformatics and Biomedicine (BIBM)*, 2025, pp 154–157.

(49) Li, Y.; Zhang, Y.; Zhu, L.; Wang, M.; Wang, R.; Wang, X. MCARSMA: A Multi-Level Cross-Modal Attention Fusion Framework for Accurate RNA–Small Molecule Affinity Prediction. *Mathematics* **2025**, *14*, 57.

(50) Wang, N.; Bian, J.; Li, Y.; Li, X.; Mumtaz, S.; Kong, L.; Xiong, H. Multi-purpose RNA language modelling with motif-aware pretraining and type-guided fine-tuning. *Nat. Mach. Intell.* **2024**, *6*, 548–557.

(51) Xie, Y.; Wang, W.; Xu, J.; Li, L.; Wu, S.; Wei, H. ncRD-LG: A Unified Framework Integrating Molecular Language Models and Subgraph Learning for Drug–ncRNA Target Prediction. *Pattern Recognition* **2026**, 113556.

(52) Tahmid, M. T.; Shahgir, H. S.; Mahbub, S.; Dong, Y.; Bayzid, M. S. BiRNA-BERT allows efficient RNA language modeling with adaptive tokenization. *Communications Biology* **2025**, *8*, 1621.

(53) Liu, X.; Li, Y.; Liu, Y.; Yuan, J.; Liu, T.; Zhou, M.; Li, J.; Zhang, Z.; Wang, X.; Ma, T., et al. PRISM: A structure-guided computational approach for identifying RNA-targeting small molecules by integrating 3D conformational and chemical information. *European Journal of Medicinal Chemistry* **2026**, 118795.

(54) Oliver, C.; Mallet, V.; Gendron, R. S.; Reinharz, V.; Hamilton, W. L.; Moitessier, N.; Waldispühl, J. Augmented base pairing networks encode RNA-small molecule binding preferences. *Nucleic Acids Res.* **2020**, *48*, note: highlight, 7690–7699.

(55) Deng, Z.; Gu, R.; Bi, H.; Ma, H.; Wang, X.; Zhang, Z.; Wen, H. In *Machine learning for structural biology workshop, NeurIPS*, 2022.

(56) Batatia, I.; Batzner, S.; Kovács, D. P.; Musaelian, A.; Simm, G. N.; Drautz, R.; Ortner, C.; Kozinsky, B.; Csányi, G. The design space of e (3)-equivariant atom-centered interatomic potentials. *arXiv* **2022**.

(57) Li, S.; Zhang, X.; Liu, M.; Zhang, H.; Jia, S.; Kang, Y.; Hou, T.; Pan, P. An accurate nucleic acid-small molecule docking framework via geometric deep learning with large-scale pretraining. *arXiv preprint arXiv:2606.05198* **2026**.

(58) Boitreaud, J.; Mallet, V.; Oliver, C.; Waldispuhl, J. OptiMol: optimization of binding affinities in chemical space for drug discovery. *J. Chem. Inf. Model.* **2020**, *60*, note: highlight, 5658–5666.

(59) Xia, J.; Zhao, C.; Hu, B.; Gao, Z.; Tan, C.; Liu, Y.; Li, S.; Li, S. Z. Mole-bert: Rethinking pre-training graph neural networks for molecules. **2023**.

(60) Ross, J.; Belgodere, B.; Chenthamarakshan, V.; Padhi, I.; Mroueh, Y.; Das, P. Large-scale chemical language representations capture molecular structure and properties. *Nat. Mach. Intell.* **2022**, *4*, 1256–1264.

(61) Huang, Z.; Fan, Z.; Shen, S.; Wu, M.; Deng, L. MolMVC: Enhancing molecular representations for drug-related tasks through multi-view contrastive learning. *Bioinformatics* **2024**, *40*, ii190–ii197.

(62) Chithrananda, S.; Grand, G.; Ramsundar, B. ChemBERTa: large-scale self-supervised pretraining for molecular property prediction. *arXiv preprint arXiv:2010.09885* **2020**.

(63) Zhou, G.; Gao, Z.; Ding, Q.; Zheng, H.; Xu, H.; Wei, Z.; Zhang, L.; Ke, G. In *The eleventh international conference on learning representations*, 2023.

(64) Galeano, D.; Imrat; Haltom, J.; Andolino, C.; Yousey, A.; Zaksas, V.; Das, S.; Baylin, S. B.; Wallace, D. C.; Slack, F. J., et al. sChemNET: a deep learning framework for predicting small molecules targeting microRNA function. *Nat. Commun.* **2024**, *15*, 9149.

(65) Grimberg, H.; Tiwari, V. S.; Tam, B.; Gur-Arie, L.; Gingold, D.; Polachek, L.; Akabayov, B. Machine learning approaches to optimize small-molecule inhibitors for RNA targeting. *J. Cheminform.* **2022**, *14*, 4.

(66) Cai, Z.; Zafferani, M.; Akande, O. M.; Hargrove, A. E. Quantitative structure–activity relationship (QSAR) study predicts small-molecule binding to RNA structure. *J. Med. Chem.* **2022**, *65*, 7262–7277.

(67) Rizvi, N. F.; Santa Maria Jr, J. P.; Nahvi, A.; Klappenbach, J.; Klein, D. J.; Curran, P. J.; Richards, M. P.; Chamberlin, C.; Saradjian, P.; Burchard, J., et al. Targeting RNA with small molecules: identification of selective, RNA-binding small molecules occupying drug-like chemical space. *SLAS Discovery* **2020**, *25*, 384–396.

(68) Yazdani, K.; Jordan, D.; Yang, M.; Fullenkamp, C. R.; Calabrese, D. R.; Boer, R.; Hilimire, T.; Allen, T. E.; Khan, R. T.; Schneekloth Jr, J. S. Machine learning informs RNA-binding chemical space. *Angew. Chem., Int. Ed.* **2023**, *135*, e202211358.

(69) Haga, C. L.; Yang, X. D.; Gheit, I. S.; Phinney, D. G. Graph neural networks for the identification of novel inhibitors of a small RNA. *SLAS Discovery* **2023**, *28*, 402–409.

(70) Xu, K.; Hu, W.; Leskovec, J.; Jegelka, S. How powerful are graph neural networks? *arXiv* **2018**.

(71) Wen, Y.; Han, Y.; Wang, D. Overcoming Shortcut Learning in RNA-Small Molecule Modeling via Bias-Matched Decoys and Structure-Aware Network Design. *Journal of Chemical Information and Modeling* **2026**, *66*, 6403–6417.

(72) Szulc, N. A.; Mackiewicz, Z.; Bujnicki, J. M.; Stefaniak, F. Structural interaction fingerprints and machine learning for predicting and explaining binding of small molecule ligands to RNA. *Briefings Bioinf.* **2023**, *24*, bbad187.

(73) Chhabra, S.; Xie, J.; Frank, A. T. RNAPosers: machine learning classifiers for ribonucleic acid–ligand poses. *J. Phys. Chem. B* **2020**, *124*, 4436–4445.

(74) Stefaniak, F.; Bujnicki, J. M. AnnapuRNA: A scoring function for predicting RNA-small molecule binding poses. *PLoS Comput. Biol.* **2021**, *17*, e1008309.

(75) Liu, Y.; Frank, A. T. Using selectively scaled molecular dynamics simulations to assess ligand poses in rna aptamers. *J. Chem. Theory Comput.* **2022**, *18*, 5703–5709.

(76) Heng Tan, L.; Keong Kwoh, C.; Mu, Y. RmsdXNA: RMSD prediction of nucleic acid-ligand docking poses using machine-learning method. *Briefings Bioinf.* **2024**, *25*, bbae166.

(77) Amburn, A.; Jayaraman Rukmani, S.; Parks, J. M.; Smith, J. C. IRIS: A Machine Learning-Based Pose Re-Ranking Tool for RNA-Ligand Docking. *bioRxiv* **2025**, 2025–10.

(78) Wang, J.; Wu, J.; Zhang, Z.; Jiang, Y.; Peng, L.; Zhang, B.; Chen, Q.; Cao, L.; Quan, L.; Lyu, Q. AffiGrapher: Contrastive Heterogeneous Graph Learning with Aromatic Virtual Nodes for RNA-Small Molecule Binding Affinity Prediction. *J. Chem. Inf. Model.* **2025**.

(79) Sun, S.; Gao, L. Contrastive pre-training and 3D convolution neural network for RNA and small molecule binding affinity prediction. *Bioinformatics* **2024**, *40*, btae155.

(80) Berman, H. M.; Westbrook, J.; Feng, Z.; Gilliland, G.; Bhat, T. N.; Weissig, H.; Shindyalov, I. N.; Bourne, P. E. The protein data bank. *Nucleic Acids Res.* **2000**, *28*, 235–242.

(81) Philips, A.; Milanowska, K.; Łach, G.; Bujnicki, J. M. LigandRNA: computational predictor of RNA–ligand interactions. *RNA* **2013**, *19*, 1605–1616.

(82) Panei, F. P.; Torchet, R.; Menager, H.; Gkeka, P.; Bonomi, M. HARIBOSS: a curated database of RNA-small molecules structures to aid rational drug design. *Bioinformatics* **2022**, *38*, 4185–4193.

(83) Krishnan, S. R.; Roy, A.; Gromiha, M. M. R-SIM: a database of binding affinities for RNA-small molecule interactions. *J. Mol. Biol.* **2023**, *435*, 167914.

(84) Passaro, S.; Corso, G.; Wohlwend, J.; Reveiz, M.; Thaler, S.; Somnath, V. R.; Getz, N.; Portnoi, T.; Roy, J.; Stark, H.; Kwabi-Addo, D.; Beaini, D.; Jaakkola, T.; Barzilay, R. Boltz-2: Towards Accurate and Efficient Binding Affinity Prediction. *bioRxiv 2025.06.14.659707* **2025**.

(85) Zhou, Y.; Jiang, Y.; Chen, S.-J. SPRank- A Knowledge-Based Scoring Function for RNA-Ligand Pose Prediction and Virtual Screening. *J. Chem. Theory Comput.* **2024**, *20*, 7358–7369.

(86) Lawson, C. L.; Berman, H. M.; Chen, L.; Vallat, B.; Zirbel, C. L. The Nucleic Acid Knowledgebase: a new portal for 3D structural information about nucleic acids. *Nucleic Acids Res.* **2024**, *52*, D245–D254.

(87) Wang, R.; Fang, X.; Lu, Y.; Yang, C.-Y.; Wang, S. The PDBbind database: methodologies and updates. *J. Med. Chem.* **2005**, *48*, 4111–4119.

(88) Townshend, B.; Kaplan, M.; Smolke, C. D. Highly multiplexed selection of RNA aptamers against a small molecule library. *PLoS One* **2022**, *17*, e0273381.
